# A20 attenuates oxidized self-DNA-mediated inflammation in acute kidney injury

**DOI:** 10.1038/s41392-025-02194-y

**Published:** 2025-04-25

**Authors:** Hanwen Li, Yongyao Wu, Lisha Xiang, Qing Zhao, Lu Liu, Zhixiong Zhu, Weimin Lin, Zhan Li, Yang Yang, Yiting Ze, Lulu Zhang, Ping Fu, Yingqiang Guo, Ping Zhang, Bin Shao

**Affiliations:** 1 State Key Laboratory of Oral Diseases & National Center for Stomatology & National Clinical Research Center for Oral Diseases, West China Hospital of Stomatology, Sichuan University, Chengdu, Sichuan PR China; 2 Division of Thoracic Tumor Multimodality Treatment and Department of Medical Oncology, Cancer Center, West China Hospital, Sichuan University, Chengdu, Sichuan PR China; 3 Department of Pediatrics, West China Second University Hospital, Sichuan University, Chengdu, Sichuan PR China; 4 Department of Biotherapy, State Key Laboratory of Biotherapy and Cancer Center, West China Hospital, Sichuan University, Chengdu, Sichuan PR China; 5 Department of Stem Cell and Regenerative Medicine, State Key Laboratory of Trauma, Burn and Combined Injury, Daping Hospital, Army Medical University, Chongqing, PR China; 6 College of Foreign Languages and Cultures, Sichuan University. Sichuan University, Chengdu, Sichuan PR China; 7 Kidney Research Institute, National Clinical Research Center for Geriatrics and Division of Nephrology, West China Hospital of Sichuan University, Chengdu, Sichuan PR China; 8 Department of Cardiovascular Surgery and Cardiovascular Surgery Research Laboratory, West China Hospital, Sichuan University, Chengdu, Sichuan PR China

**Keywords:** Inflammation, Cell biology

## Abstract

The ubiquitin-editing enzyme A20 is known to regulate inflammation and maintain homeostasis, but its role in self-DNA-mediated inflammation in acute kidney injury (AKI) is not well understood. Here, our study demonstrated that oxidized self-DNA accumulates in the serum of AKI mice and patients. This oxidized self-DNA exacerbates the progression of AKI by activating the cGAS-STING pathway and NLRP3 inflammasome. While inhibition of the STING pathway only slightly attenuates AKI progression, suppression of NLRP3 inflammasome-mediated pyroptosis significantly alleviates AKI progression and improves the survival of AKI mice. Subsequently, we found that *Tnfaip3* (encoding A20) is significantly upregulated following oxidized self-DNA treatment. A20 significantly alleviates AKI development by dampening STING signaling pathway and NLRP3-mediated pyroptosis. Moreover, A20-derived peptide (P-II) also significantly alleviates ox-dsDNA-induced pyroptosis and improves the survival and renal injury of AKI mice. Mechanistically, A20 competitively binds with NEK7 and thus inhibiting NLRP3 inflammasome. A20 and P-II interfere with the interaction between NEK7 and NLRP3 through Lys140 of NEK7. Mutation of Lys140 effects on the interaction of NEK7 with A20 and/or NLRP3 complex. Conditional knockout of NEK7 in macrophages or pharmacological inhibition of NEK7 both significantly rescue AKI mouse models. This study reveals a new mechanism by which A20 attenuates oxidized self-DNA-mediated inflammation and provides a new therapeutic strategy for AKI.

## Introduction

Acute kidney injury (AKI) is a life-threatening condition characterized by a rapid loss of renal function, leading to high mortality and morbidity.^1,2^ Approximately 13% of patients received the first course of cisplatin treatment developed AKI.^2,3^ Despite various initial triggers, the pathophysiology of AKI often involves common pathogenic denominators, such as cell death, tissue injury, and drastic inflammation.^4^ Several types of cell death have been recently described in AKI, and multiple strategies targeting cell death have been reported to help mitigate the progression of AKI.^5,6^ Danger-associated molecular patterns (DAMPs) and proinflammatory factors are released from dying cells uncontrollably, leading to secondary cell death and further amplifies tissue injury.^7^

As DAMPs, self-DNA originates from dying cells and is present in pathological conditions such as cancer, trauma, and viral infection.^8^ Self-DNA primarily consists of mitochondrial DNA, endogenous retroelements, and chromosomal DNA. An increased cytoplasmic DNA load is associated with immune recognition and sterile inflammation.^9–11^ Cytosolic double-stranded DNA (dsDNA) can be recognized by both the cGAS-STING pathway and the absence in melanoma 2 (AIM2) inflammasome. Activation of the cGAS-STING pathway motivates antiviral immunity by upregulating type I interferon (IFN), while AIM2 activation leads to gasdermin-mediated lytic programmed cell death named pyroptosis and the subsequent secretion of cytokines such as IL-1β and IL-18.^12–14^ Interestingly, oxidized dsDNA (ox-dsDNA) can specifically enable the nucleotide-binding domain and leucine rich repeat (NLR) pyrin domain containing 3 (NLRP3) inflammasome complex, rather than AIM2, to accelerate inflammation and tissue damage. This form of dsDNA shows low susceptibility to exonuclease, making it resistant to extracellular degradation.^15,16^ This resilient form of dsDNA is capable of potentiating the cytosolic immune response, such as via the activation of STING signaling.^15^ Moreover, the highly activated cGAS-STING axis can further promote mobilization of the NLRP3 inflammasome in human myeloid cells, independently of AIM2.^17^ Collectively, this evidence underscores the significant role of the NLRP3 inflammasome in mediating ox-DNA-induced inflammation.

NLRP3 is a cytosolic sensor of cellular perturbations and environment irritants, acting as a crucial integration point for various stimuli and sensing dyshomeostatic cellular state.^18,19^ Activation of NLRP3 leads to assembly of apoptosis-associated speck-like protein containing a caspase recruitment domain (ASC) speck and formation of molecular platform comprising NLRP3, ASC and pro-IL-1β.^20^ This assembly and subsequent activation of NLRP3 inflammasome induce caspase-1-mediated cleavage of gasdermin D (GSDMD), resulting in release of cellular contents and pyroptosis.^21^ NLRP3 is activated by various microbial and sterile stimuli, including bacterial toxins, extracellular ATP, and oxidated DNA.^20,22^ A critical step in this activation process is potassium efflux, which is essential for NLRP3 inflammasome activation. Downstream of potassium efflux, NIMA-related kinase 7 (NEK7), a serine/threonine kinase, directly binds NLRP3, regulating its subsequent oligomerization and activation.^23^ Therefore, NEK7 represents a promising target for diseases mediated by NLRP3 inflammasome activation.

As a ubiquitin-editing enzyme, A20 contains diverse domains that facilitate both ubiquitination and deubiquitination.^24,25^ Previous studies suggested that A20 modulates the activation of the NLRP3 inflammasome through multiple mechanisms. A20 can restrict NLRP3’s transcriptional synthesis by inhibiting the NF-κB signaling pathway.^26,27^ A20 curbs the spontaneous activation of NLRP3 by limiting the K63-linked ubiquitination of the pro-IL-1β complex.^28^ Additionally, A20 possesses non-catalytic ubiquitin-binding functions, which can significantly inhibit the spontaneous activation of NLRP3.^29,30^ Negative regulation of the NLRP3 inflammasome in macrophages by A20 protects mice against inflammatory diseases, such as arthritis^27^ and sepsis.^29^ Notably, reducing A20’s enzymic anti-inflammatory function results in increased stimulus-dependent NF-κB activation and subsequent deterioration of AKI.^31^ Whereas, the specific role of A20 in regulating AKI has not been elucidated yet.

Our study demonstrates that oxidized self-DNA released from dying cells contributes to the progression of AKI by mediating inflammation, which primarily relies on pyroptosis and partially depends on the cGAS-STING pathway. A20 attenuates AKI by dampening these signaling pathways. Specifically, our results suggest that A20 mitigates NLRP3 activation by interacting with the NEK7 protein, thereby interfering with its binding to the NLRP3 complex. This interference attenuates the ox-DNA-NEK7-NLRP3-pyroptosis axis-induced inflammation. Consequently, this study provides a theoretical basis for the clinical treatment of AKI.

## Results

### Oxidized self-DNA promotes the progression of AKI

**Fig. 1:**
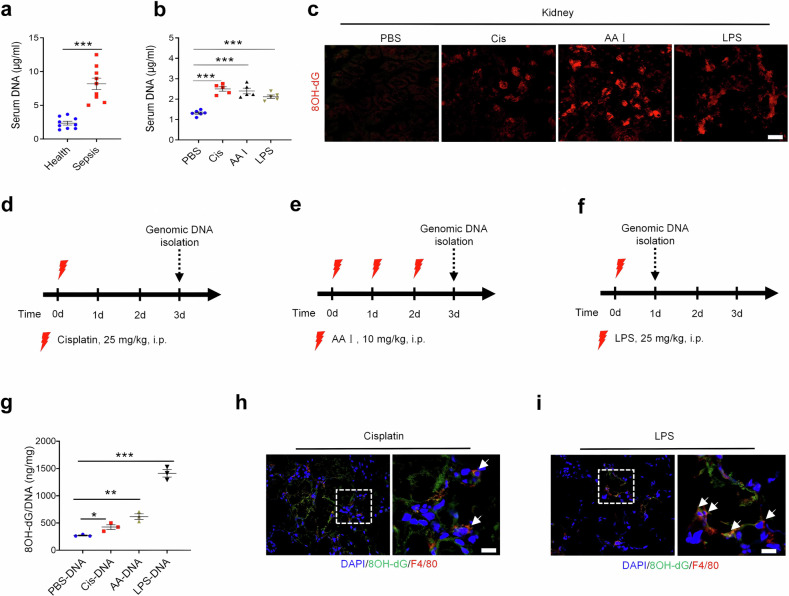
Self-DNA is oxidized and released in AKI. **a** Serum DNA value of healthy donors and active sepsis patients. (*n* = 9, mean ± SEM); ***P < 0.001. **b** Serum DNA values of mice treated with PBS, Cis, AA I, or LPS. (*n* = 5, mean ± SEM); ***P < 0.001. **c** Fluorescence microscopy of 8OH-dG (red) in kidneys of mice that were treated with PBS, Cis, AA I, or LPS (*n* = 3). scale bars, 10 μm. Schematic of genomic DNA isolation of renal tissue from mice that were intraperitoneally injected with cisplatin (**d**) (named Cis-DNA), AA I (**e**) (AA-DNA) or LPS (**f**) (LPS-DNA), and the specific time of implementing isolation was indicated in timeline. **g** 8OH-dG concentrations in DNA extracted from renal tissue of PBS, Cis, AA I, and LPS treated mice (*n* = 3, mean ± SEM); *P < 0.05, **P < 0.01, ***P < 0.001. **h** Fluorescence microscopy of DAPI (blue), 8OH-dG (green), and F4/80 (red) in kidneys of mice treated with Cis (*n* = 3). scale bars, 10 μm. **i** Fluorescence microscopy of DAPI (blue), 8OH-dG (green), and F4/80 (red) in kidneys of mice treated with LPS (*n* = 3). scale bars, 10 μm

To investigate whether there is self-DNA release in AKI and considering that sepsis is one of the common causes of AKI, we collected human serum samples from sepsis patients and normal donors, and patients suffering from sepsis were found to exhibit increased serum DNA levels (Fig. 1a). Meanwhile, AKI mouse models were created by injection with cisplatin, aristolochic acid I (AA I) or lipopolysaccharide (LPS). As expected, these AKI mice demonstrated obviously elevated levels of DNA in serum accompanied by dramatically increased 8-hydroxy-2′-deoxyguanosine (8OH-dG) in renal tissue (Fig. 1b, c). Subsequently, normal renal tissue and kidneys of cisplatin-, AA I- or LPS-induced AKI mice were collected to implement genomic DNA purification (named PBS-DNA, Cis-DNA, AA-DNA, and LPS-DNA hereafter) (Fig. 1d–f), and increased 8OH-dG levels were detected in mice treated with Cis-DNA, AA-DNA, and LPS-DNA in comparison with PBS-DNA-treated mice (Fig. 1g). Macrophages are defined as the main effector cells and a druggable target for acute kidney inflammation.^32,33^ Moreover, DAMPs, such as endogenic DNA from dying cells, can be recognized by macrophages to motivate immune response.^34,35^ Indeed, our data from immunofluorescent staining suggested that ox-self-DNA was engulfed by macrophages in the renal tissue of cisplatin- and LPS-treated mice (Fig. 1h, i). Therefore, macrophages might further amplify inflammation and tissue damage through activation of ox-self-DNA-induced signaling pathways.

### Oxidized self-DNA release activates STING signaling pathway and promoting the development of AKI

**Fig. 2:**
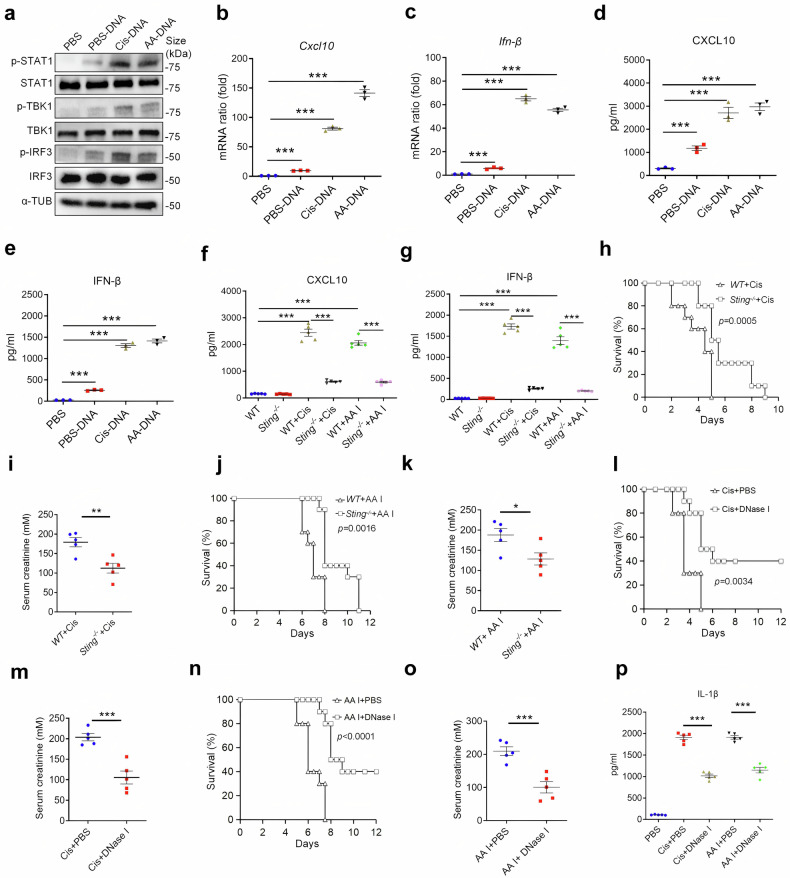
Ox-self-DNA promotes the progression of AKI by activating STING signaling pathway. **a** Immunoblotting showing expression levels of indicated proteins in BMDMs treated with PBS, PBS-DNA, Cis-DNA, or AA-DNA. Quantitative PCR analysis of *Cxcl10* (**b**) and *Ifn-β* (**c**) mRNA in BMDMs treated with PBS, PBS-DNA, Cis-DNA, or AA-DNA. The expression of *Gapdh* mRNA was used to normalize the results. (*n* = 3, mean ± SEM); ***P < 0.001. **d**, **e** CXCL10 (**d**) and IFN-β (**e**) levels from supernatants of BMDMs stimulated with PBS, PBS-DNA, Cis-DNA, or AA-DNA. (*n* = 3. mean ± SEM); ***P < 0.001. Expression levels of serum CXCL10 (**f**) and IFN-β (**g**) in WT and *Sting*^*−/−*^ mice intraperitoneally injected with PBS, Cis, or AA I were measured by ELISA. (*n* = 5, mean ± SEM); ***P < 0.001. **h** Survival of WT mice and *Sting*^*−/−*^ mice intraperitoneally injected with Cis. (*n* = 10 per group). **i** Serum creatinine level of mice as described in (**h**). (*n* = 5. mean ± SEM); **P < 0.01. **j** Survival of WT mice and *Sting*^*−/−*^ mice intraperitoneally injected with AA I. (*n* = 10 per group). **k** Serum creatinine level of mice as described in (**j**). (*n* = 5. mean ± SEM); *P < 0.05. **l** Survival of Cis plus PBS or Cis plus DNase I treated mice. (*n* = 10 per group). **m** Serum creatinine level of mice as described in (**l**). (*n* = 5. mean ± SEM); ***P < 0.001. **n** Survival of AA I plus PBS or AA I plus DNase I treated mice. (*n* = 10 per group). **o** Serum creatinine level of mice as described in (**n**) (*n* = 5. mean ± SEM); ***P < 0.001. **p** Serum level of IL-1β in mice with pharmaceutical employment as described in (**l**, **n**). (*n* = 5, mean ± SEM); ***P < 0.001

Previous study has reported that cGAS-STING pathway is capable of sensing and regulating the cellular response towards the host-derived DNAs.^36^ We further determine whether oxidized self-DNA (ox-self-DNA) aggravates AKI by activating STING signaling pathway. Our data suggested that STING signaling pathway related proteins, such as phosphorylated activator of transcription 1 (p-STAT1), phosphorylated TANK Binding Kinase 1 (p-TBK1) and phosphorylated interferon regulatory factor 3 (p-IRF3), were significantly increased in renal tissue of cisplatin-treated mice (Supplementary Fig. 1a), along with elevated mRNA levels of proinflammatory cytokines, including *Ifn-β*, *Tnf-α* and *Il-6* (Supplementary Fig. 1b-d). We next measured the levels of STING-related proteins in BMDMs treated with PBS, PBS-DNA, Cis-DNA or AA-DNA. Consistent with our in vivo data and previous studies,^37^ the expression levels of STING signaling pathway related proteins were significantly increased after ox-self-DNA stimulation (Fig. 2a). Correspondingly, mRNA levels of *Cxcl10* and *Ifn-β* were elevated (Fig. 2b, c), accompanied by excessive secretion of CXCL10 and IFN-β into the culture medium (Fig. 2d, e). To further validate the role of STING pathway in AKI, we used *Sting*-knockout mice (*Sting*^*−/−*^) and stimulated them with cisplatin and AA I. As a result, *Sting*^*−/−*^ mice exhibited lower elevated secretion of CXCL10 and IFN-β (Fig. 2f, g), and showed improved survival and decreased serum creatinine compared to wild-type (WT) mice, despite treatment with AA I and cisplatin (Fig. 2h–k). However, inhibition of the STING pathway only moderately improved the survival and attenuated kidney injury in vivo (Supplementary Fig. 1e-h). Considering that DNase I is a widely used enzyme in the therapy of cell free DNA (cfDNA)-related diseases,^6,38^ we employed DNase I to determine its efficiency in attenuating AKI. Surprisingly, the therapeutic effect of DNase I was more pronounced compared to that in *Sting*^*−/−*^ mice (Fig. 2l–o; Supplementary Fig. 1i-l). Additionally, DNase I significantly reduced serum level of IL-1β in AKI mice (Fig. 2p). Therefore, ox-self-DNA aggravates the development of AKI partially through the activation of STING pathway, suggesting that other signaling pathways may also contribute to ox-self-DNA-induced progression of AKI.

### Oxidized self-DNA release motivated pyroptosis of macrophage and promoting the development of AKI

**Fig. 3:**
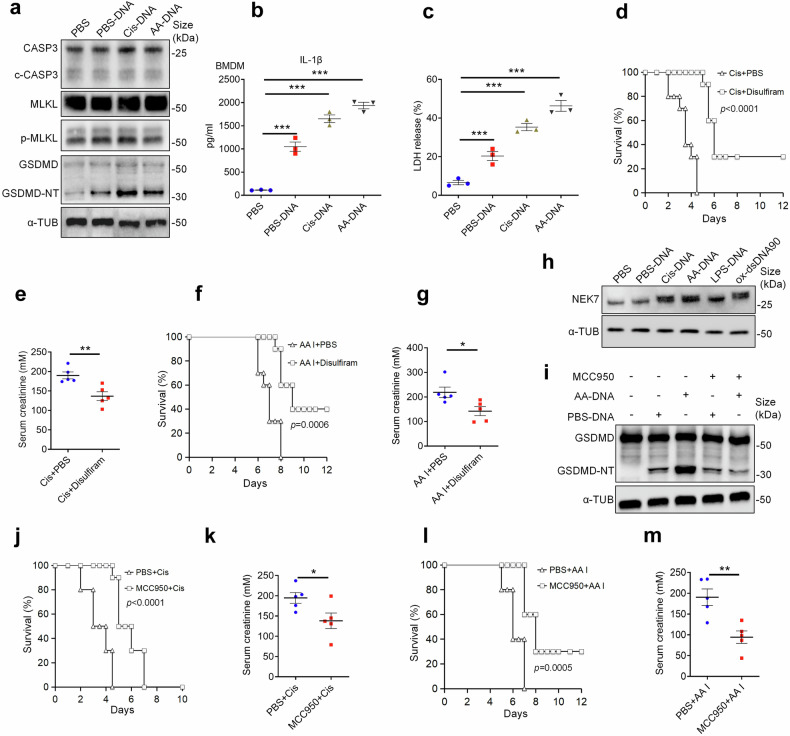
Ox-self-DNA promotes the progression of AKI by facilitating pyroptosis. **a** Immunoblots for total and cleaved caspase-3, MLKL, and phosphorylated MLKL, total and cleaved GSDMD from BMDMs treated with PBS, PBS-DNA, Cis-DNA, or AA-DNA. IL-1β level of supernatant from BMDMs treated with PBS, PBS-DNA, Cis-DNA, or AA-DNA was analyzed by ELISA (**b**), with cell death determined by LDH release (**c**), (*n* = 3, mean ± SEM); ***P < 0.001. **d** Survival of mice injected with Cis plus PBS or Cis plus disulfiram (*n* = 10 per group). **e** Serum creatinine level of mice as described in **d**. (*n* = 5. mean ± SEM); **P < 0.01. **f** Survival of mice injected with AA I plus PBS or AA I plus disulfiram (*n* = 10 per group). **g** Serum creatinine level of mice as described in (**f**). (*n* = 5. mean ± SEM); *P < 0.05. **h** Immunoblots for NEK7 from BMDMs treated with PBS or various DNA. **i** Immunoblots for total and cleaved GSDMD from iBMDMs treated with PBS-DNA or AA-DNA with or without MCC950 treatment. **j** Survival of mice treated with Cis and intraperitoneally preinjected with PBS or MCC950 (*n* = 10 per group). **k** Serum creatinine level of mice as described in (**j**). (*n* = 5. mean ± SEM); *P < 0.05. **l** Survival of mice treated with AA I and intraperitoneally preinjected with PBS or MCC950 (*n* = 10 per group). **m** Serum creatinine level of mice as described in (**l**). (*n* = 5. mean ± SEM); **P < 0.01

Previous studies have reported that the release of cytosolic oxidized mitochondrial DNA specifically activates the NLRP3 inflammasome,^16^ and activation of NLRP3 is closely related to pyroptosis. We further investigated whether ox-self-DNA release promotes pyroptosis in cisplatin-induced AKI. Consistently, our data showed that self-DNA treated BMDMs exhibited significantly increased level of N-terminal pore-forming GSDMD fragment (GSDMD-NT), a key pyroptotic executioner (Fig. 3a), along with elevated release of IL-1β and LDH (Fig. 3b, c). Notably, ox-self-DNA (Cis-DNA and AA-DNA) demonstrated a superior capability in inducing pyroptosis compared to PBS-DNA. In vivo, AKI mice pretreated with the GSDMD inhibitor disulfiram showed improved survival with effects comparable to those of DNase I (Fig. 3d–g; Supplementary Fig. 2a-d). Interestingly, the expression level of NLRP3 upstream regulatory molecule NEK7 increased under various ox-self-DNA and ox-dsDNA90 (positive control) conditions (Fig. 3h). Moreover, the pharmacological inhibitor of the NLRP3 signaling pathway, MCC950, significantly reduced the cleavage of GSDMD after ox-self-DNA treatment (Fig. 3i), and MCC950 treatment also improved the survival and attenuate tubular injury of mice treated with nephrotoxic drugs (Fig. 3j-m; Supplementary Fig. 2e-h). Thus, the NLRP3 inflammasome plays a crucial role in the ox-self-DNA-promoted progression of AKI.

### A20 is a key molecule in response to ox-self-DNA stimulation

**Fig. 4:**
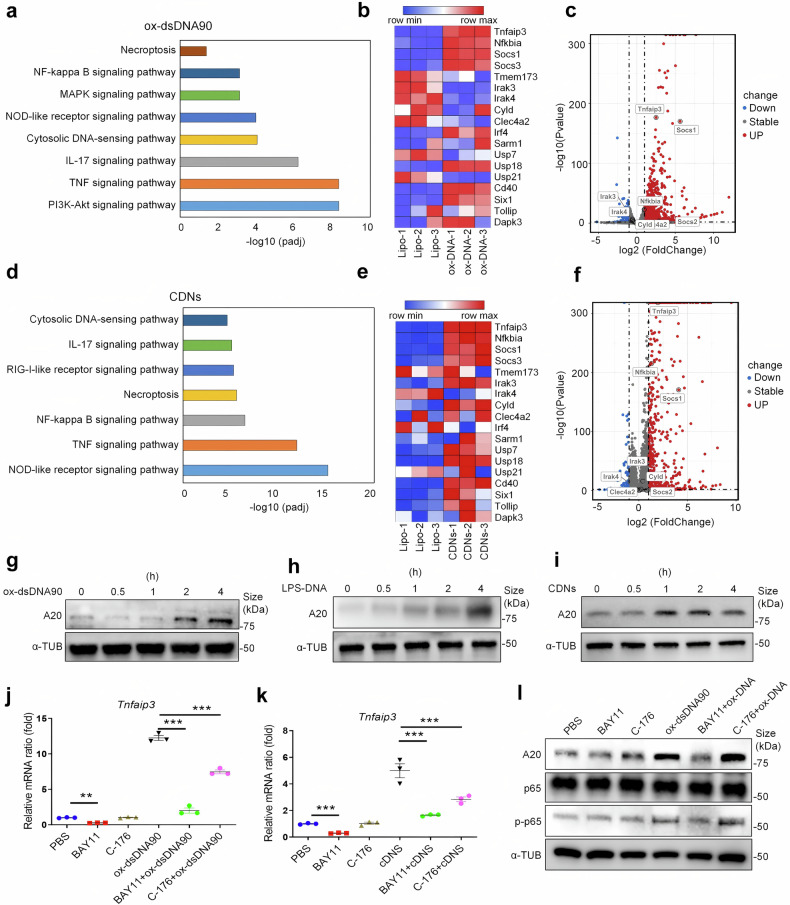
A20 is a key molecule in response to ox-DNA. RNA-Seq was performed on mRNA isolated from ox-dsDNA90- or CDNs-treated BMDMs. **a**, **d** Kyoto Encyclopedia of Genes and Genomes (KEGG) pathway analysis demonstrated the upregulated pathways. Heatmaps of differentially expressed genes reactive to ox-dsDNA90 (**b**) or CDNs (**e**) stimulation. Volcano Plot of differential gene expression of ox-dsDNA90- (**c**) or CDNs- (**f**) treated BMDMs. Immunoblots for A20 from iBMDMs respectively treated with ox-dsDNA90 (**g**), LPS-DNA (**h**) or CDNs (**i**). **j** Quantitative PCR analysis of *Tnfaip3* mRNA in iBMDMs treated with PBS, BAY11-7082 (1 μM), C-176 (1 μM), ox-dsDNA90, ox-dsDNA90 along with BAY11-7082 (1 μM, 4 h pretreated before ox-dsDNA90 stimulation) or ox-dsDNA90 along with C-176 (1 μM, 24 h pretreated before ox-dsDNA90 stimulation). (*n* = 3. mean ± SEM); **P < 0.01, ***P < 0.001. **k** Quantitative PCR analysis of *Tnfaip3* mRNA in iBMDMs treated with indicated reagents. (*n* = 3, mean ± SEM); ***P < 0.001. **l** Immunoblots for A20, NF-κB-p65, and phosphorylated NF-κB-p65 from iBMDMs treated as described in (**j**)

As previous studies have indicated, acute inflammation is typically self-limiting and diminishes following the removal of noxious stimuli.^39^ AKI is commonly recognized as acute inflammation condition, generally confined to a duration of seven days.^38^ Therefore, it is crucial to investigate whether there is a self-limiting mechanism or molecule that exists to mediate the anti-inflammatory function. To identify targets that might restrict the exaggerated inflammation resulting from ox-self-DNA, we performed RNA sequencing (RNA-Seq) to examine transcriptome profiles in BMDMs treated with ox-dsDNA90. Kyoto Encyclopedia of Genes and Genomes (KEGG) pathway analysis was then used to determine the activated pathway. As expected, the cytosolic DNA-sensing pathway, NOD-like receptor signaling pathway, and NF-κB signaling pathway were highly enriched in the oxidized DNA-treated group (Fig. 4a). To identify the potential self-limiting mechanism, we further investigated canonical molecules known to participate in these pathways through document retrieval and screened for the significantly elevated molecules in response to oxidized DNA as a consequence of RNA-Seq. Notably, oxidized DNA stimulation led to significant upregulation of *Tnfaip3* (encoding A20) (Fig. 4b, c), a key regulator of inflammation, immunity, and homeostasis.^40^ Additionally, significant upregulation of *Tnfaip3* was also observed in BMDMs stimulated with cyclic dinucleotides (CDNs, second messenger that could activate STING pathway), and KEGG pathway analysis also demonstrated that NOD-like receptor signaling pathway and NF-κB signaling pathway were highly enriched in the CDNs-treated group (Fig. 4d-f). These data indicated a potential link between STING signaling pathway and NLRP3 inflammasome, consistent with the previous study which indicated NLRP3 could be activated downstream of the STING pathway.^17^

Based on the above inference, BMDMs were cultured and stimulated with ox-dsDNA90, LPS-DNA, and CDNs. Our results indicated that the expression level of A20 increased with time (Fig. 4g-i). Considering that A20 is an NF-κB-induced molecule, we further investigated whether the increased expression of A20 was connected to the activation of the NF-κB or STING signaling pathways. Cells were treated with the NF-κB pathway inhibitor BAY11-7082 or the STING signaling pathway inhibitor C-176, followed by stimulation with ox-dsDNA90 or CDNs. Notably, treatment with BAY11-7082 almost completely inhibited the increased expression of A20 at both the mRNA and protein levels. However, C-176 had a limited effect on the protein level of A20 and only partially impacted its mRNA level (Fig. 4j-l). These findings suggest that A20 is an important reactive molecule in response to stimulation by oxidized DNA or CDNs, and its expression is highly dependent on the management of NF-κB.

### A20 restricts ox-self-DNA-induced activation of STING signaling pathway

**Fig. 5:**
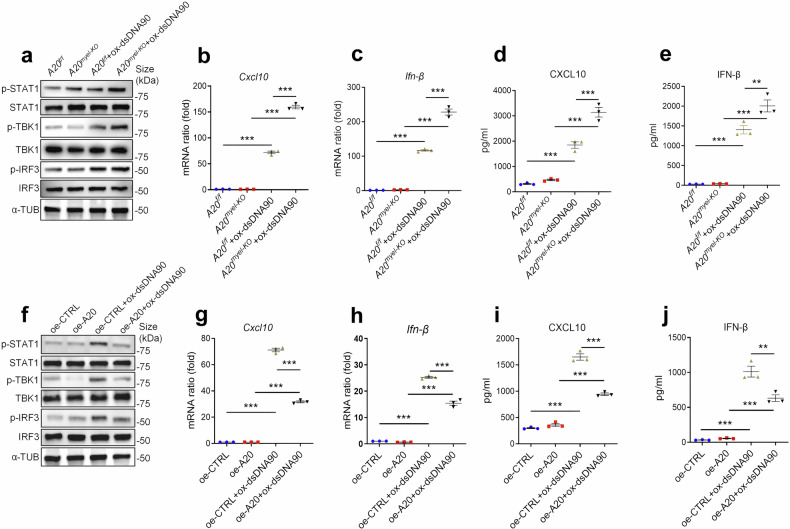
A20 inhibits STING activation. **a** Immunoblots for STAT1 and phosphorylated STAT1, TBK1 and phosphorylated TBK1, IRF3 and phosphorylated IRF3 from *A20*^*f/f*^ BMDMs and *A20*^*myel-KO*^ BMDMs with or without ox-dsDNA90 treatment. Quantitative PCR analysis of *Cxcl10* (**b**) and *Ifn-β* (**c**) mRNA in BMDMs isolated from *A20*^*f/f*^ BMDMs and *A20*^*myel-KO*^ BMDMs with or without treatment with ox-dsDNA90. (*n* = 3, mean ± SEM); ***P < 0.001. CXCL10 (**d**) and IFN-β (**e**) ELISA of supernatant from BMDMs as described in (**a**). (*n* = 3, mean ± SEM); **P < 0.01, ***P < 0.001. **f** Immunoblots for STAT1 and phosphorylated STAT1, TBK1, and phosphorylated TBK1, IRF3 and phosphorylated IRF3 from BMDMs infected with lentivirus encoding green fluorescent protein (GFP) (oe-CTRL) or mouse A20/GFP (oe-A20) with or without ox-dsDNA90 treatment. Quantitative PCR analysis of *Cxcl10* (**g**) and *Ifn-β* (**h**) mRNA in oe-CTRL iBMDMs and oe-A20 iBMDMs with or without treatment with ox-dsDNA90. (*n* = 3, mean ± SEM); ***P < 0.001. CXCL10 (**i**) and IFN-β (**j**) ELISA of supernatant from oe-CTRL iBMDMs or oe-A20 iBMDMs treated as indicated in (**c**). (*n* = 3, mean ± SEM); **P < 0.01, ***P < 0.001

To delete A20 in myeloid cells, we generated mice with a specific deletion of A20 in myeloid cells (*A20*^*myel-KO*^) by crossing the A20 flox (*A20*^*f/f*^) mice into lysozyme M (LysM)-Cre-recombinase-expressing mice. *A20*^*myel-KO*^ macrophages exhibited elevated expression of protein related to STING signaling pathway (Fig. 5a). Our data also suggested that *A20*^*myel-KO*^ cells were more sensitive to ox-dsDNA90 stimulation, showing elevated mRNA level of *Cxcl10* and *Ifn-β* accompanied by increased expression and secretion of CXCL10 and IFN-β (Fig. 5b-e). Conversely, overexpressing A20 in immortalized BMDMs (oe-A20-iBMDMs) resulted in decreased STING pathway activation compared with oe-CTRL-iBMDMs upon ox-dsDNA90 stimulation (Fig. 5f). Moreover, overexpression of A20 in iBMDMs enhanced their resistance to ox-dsDNA90 stimulation, as demonstrated by significantly decreased expression level of *Cxcl10* and *Ifn-β* and reduced secretion of corresponding cytokines compared to oe-CTRL-iBMDMs (Fig. 5g–j). These data indicate that A20 can attenuate ox-self-DNA-induced activation of the STING signaling pathway.

### A20 restricts ox-self-DNA-induced pyroptosis and attenuates the progression of AKI

**Fig. 6:**
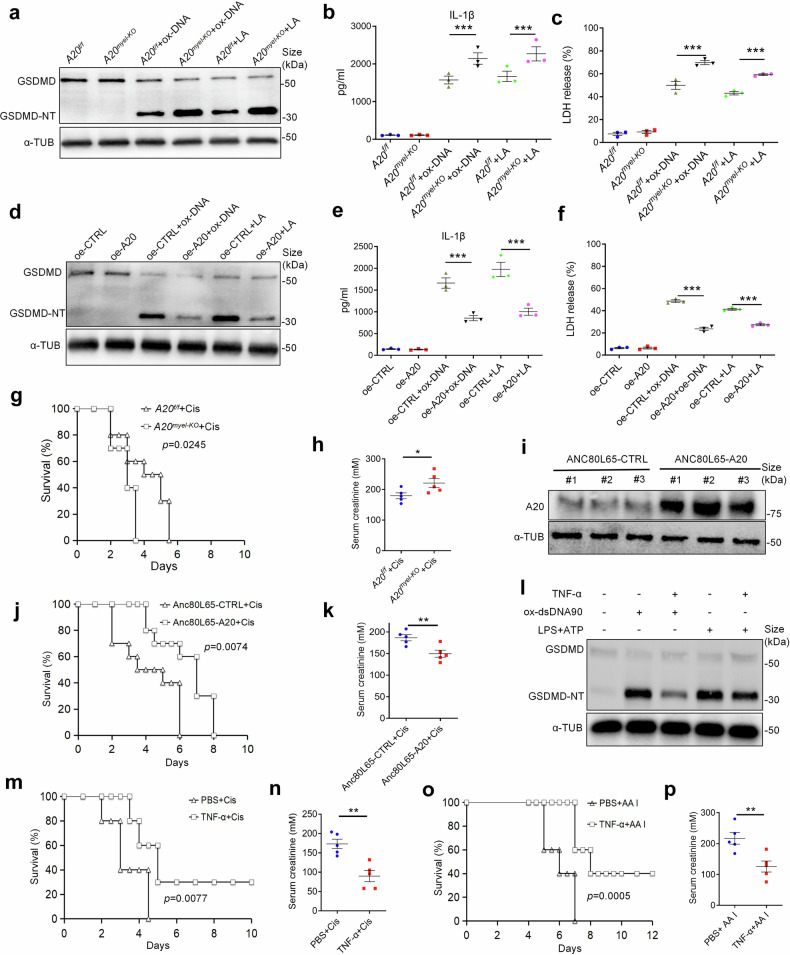
A20 inhibits ox-DNA-induced pyroptosis and limits the progression of AKI. **a** BMDMs was isolated from *A20*^*f/f*^ or *A20*^*myel-KO*^ mice and then stimulated with PBS, ox-dsDNA90 (ox-DNA) or LPS plus ATP (LA). The total and cleaved GSDMD of these BMDMs were measured by immunoblotting. IL-1β (**b**) and LDH (**c**) levels from the supernatant of BMDMs as depicted in (**a**) were measured. (*n* = 3, mean ± SEM). ***P < 0.001. **d** Immunoblots for total and cleaved GSDMD from PBS, ox-dsDNA90 or LPS followed by ATP stimulated oe-CTRL iBMDMs or oe-A20 iBMDMs. IL-1β (**e**) level from the supernatant of iBMDMs as described in (**d**) were measured by ELISA, and cell death was determined by LDH (**f**). (*n* = 3, mean ± SEM). ***P < 0.001. **g** Survival of Cis treated *A20*^*f/f*^ or *A20*^*myel-KO*^ mice. (*n* = 10 per group). **h** Serum creatinine of Cis-treated *A20*^*f/f*^ or *A20*^*myel-KO*^ mice. (*n* = 5, mean ± SEM). *P < 0.05. **i** Immunoblots for A20 from renal tissue of mice treated with Anc80L65-CTRL or Anc80L65-A20 (# indicates the serial number of individual mouse). **j** Survival of mice treated with Anc80L65-CTRL plus Cis or Anc80L65-A20 plus Cis. (*n* = 10 per group). **k** Serum creatinine of mice as depicted in (**j**). (*n* = 5, mean ± SEM). **P < 0.01. **l** Immunoblots for total and cleaved GSDMD from BMDMs stimulated with PBS, ox-dsDNA90, or LPS followed by ATP with or without pretreatment of TNF-α. **m** Survival of mice treated with Cis, with or without pretreatment of TNF-α. (*n* = 10 per group). **n** Serum creatinine of mice as depicted in (**m**). (*n* = 5, mean ± SEM). **P < 0.01. **o** Survival of mice treated with AA I, with or without pretreatment of TNF-α. (*n* = 10 per group). **p** Serum creatinine of mice as depicted in (**o**). (*n* = 5, mean ± SEM). **P < 0.01

Additionally, we investigated whether A20 restricts ox-self-DNA-induced pyroptosis. Our result suggested that *A20*^*myel-KO*^ macrophages exhibited increased expression of the pyroptotic executioner protein and the proinflammatory cytokine IL-1β when treated with ox-dsDNA90 or LPS plus ATP, along with increased LDH release (Fig. 6a-c). In contrast, overexpression of A20 in immortalized BMDMs (oe-A20-iBMDMs), achieved using lentivirus, rendered the cells more resistant to ox-dsDNA90 and LPS plus ATP stimulation, resulting in reduced pyroptosis, lower IL-1β secretion, and decreased LDH release (Fig. 6d–f). In vivo, *A20*^*myel-KO*^ mice demonstrated shorter survival times and lower survival rates than *A20*^*f/f*^ mice after AKI induction (Fig. 6g), accompanied by increased serum creatinine and aggravated tubular injury (Fig. 6h; Supplementary Fig. 3a, b). Conversely, overexpression of A20 in renal tissue by employing adeno-associated virus (AAV) Anc80L65 provided a modest survival benefit to AKI mice (Fig. 6i, j). Moreover, Anc80L65-treated mice demonstrated decreased serum creatinine and alleviated tubular injury (Fig. 6k; Supplementary Fig. 3c, d). Additionally, as TNF-α was reported to promote the expression of A20,^41^ we pretreated BMDMs with low-dose TNF-α before ox-self-DNA or LPS plus ATP stimulation, and injected mice with low-dose TNF-α prior to cisplatin and AA I injection. As expected, BMDMs pretreated with TNF-α were more resistant to pyroptotic induction than their counterparts (Fig. 6l). In vivo, the AKI mice with TNF-α pre-injection exhibited extended overall survival benefit, as well as alleviated serum creatinine and tubular injury (Fig. 6m-p; Supplementary Fig. 3e-h). These results indicate that A20 restricts ox-self-DNA-induced pyroptosis and STING pathway activation, thereby attenuating AKI.

### A20 attenuates ox-self-DNA-mediated pyroptosis through disrupting binding of NEK7 and NLRP3 complex

**Fig. 7:**
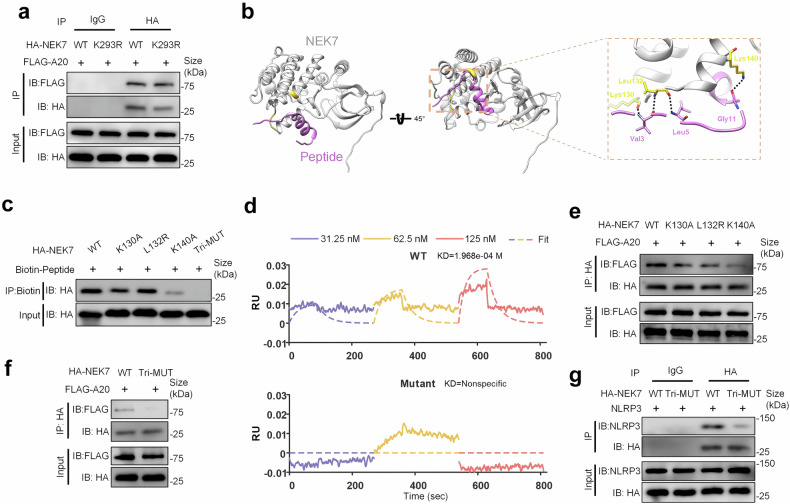
A20 binds with NEK7 and impedes the interaction of NEK7 and NLRP3. **a** HEK293T were transiently transfected with FLAG-A20 plasmids and indicated NEK7 associated/derived plasmids, including WT-NEK7 and individual mutation of K293 of NEK7. The protein level of FLAG-A20 in immunoprecipitates of NEK7 was detected by immunoblotting. **b** The predicted structure of NEK7 (light gray) and peptide P-II (orchid). Coordinates of Lys130, Leu132 and Lys140 (gold) of NEK7 interact with Val3, Leu5 and Gly11 of peptide, respectively. Dashed lines indicate hydrogen bonds. **c** HEK293T were transiently transfected with indicated HA-NEK7 associated/derived plasmids, including WT, individual mutation of K130, L132 or K140, and simultaneous mutation of K130, L132, and K140. The protein level of HA-NEK7 in immunoprecipitates of biotin-P-II was detected by immunoblotting. Abbreviation: Tri-MUT is a triple mutation. **d** P-II were dissolved with ddH_2_O and diluted into the indicated concentration. HA-WT-NEK7 or HA-Tri-MUT-NEK7 plasmids were transfected into HEK293T and purified by immunoprecipitation. The kinetic interaction of NEK7 and P-II was detected by BLI analysis. Fitting curve was shown as a dotted line. **e**, **f** HEK293T were transiently transfected with FLAG-A20 and indicated HA-NEK7 associated/derived plasmids. The protein level of FLAG-A20 in immunoprecipitates of HA-NEK7 was detected by immunoblotting. **g** FLAG-A20, NLRP3, and indicated plasmids of HA-NEK7 were transiently transfected into HEK293T. Immunoprecipitates were immunoblotted with the indicated antibodies

Our previous data demonstrated that A20 is capable of binding to NEK7 thus promoting its proteasomal degradation.^29^ Moreover, our data from mass spectrometry identified that binding between A20 and NEK7 was more pronounced under the stimulation of ox-DNA (Supplementary Fig. 4a). In addition, the OTU domain, ZnF4 domain, and ZnF7 domain of A20 are crucial for its integration with NEK7 (Supplementary Fig. 4b-d). However, the specific site of NEK7 that binding with A20 is not clear. Therefore, we predicted the potential binding sites of A20 and NEK7 by using molecular docking. The result indicated several sites of NEK7 that might support its binding to A20 (Supplementary Fig. 5a-d). Among these, the ubiquitinated lysine K293 has been proven to play a crucial role in mediating the ubiquitination and degradation of NEK7 by A20. Whereas, lysine (K) to arginine (R) mutation of NEK7’s K293 has no impact on the binding of A20 and NEK7 (Fig. 7a).

Previously, we generated a NEK7 targeting peptide (P-II) according to the OTU domain of A20, which displayed a high affinity for NEK7 and has the capability of inhibiting ox-self-DNA-induced pyroptosis (Supplementary Fig. 4e-i). Considering the flexible structure and motion of these two protein conformations, we predicted the potential binding sites between P-II and NEK7 using molecular docking (Fig. 7b). The result suggested Lys130, Leu132, and Lys140 were the potential residues that have roles in substrate binding of NEK7 and P-II. Next, we generated point mutations of NEK7: including lysine (K) to alanine (A) mutation of Lys130 (K130A), leucine (L) to arginine (R) mutation of Leu132 (L132R), lysines (K) to alanine (A) mutation of Lys140 (K140A), and simultaneous mutation of three residues. Then, we performed co-IP on varied NEK7 by using biotin-labeled P-II. Our results suggested that K140A mutant and simultaneous mutation of three residues could significantly attenuate the binding of NEK7 and P-II (Fig. 7c). In addition, result from BLI analysis also showed that simultaneous mutation of these three residues abrogated the affinity of NEK7 for P-II (Fig. 7d). We further explored whether these point mutations of NEK7 have an effect on its binding to A20 protein. Consistent with peptide data, our co-IP experiment demonstrated that K140A mutant have significant impact on the binding of A20 and NEK7, while K130A and L132R does not seem to affect the binding of the two proteins (Fig. 7e). Additionally, simultaneous mutation of these three residues also abrogated this binding (Fig. 7f). Interestingly, simultaneous mutation of K130, L132, and K140 in NEK7 protein abolished the binding of NEK7 to NLRP3 complex (Fig. 7g). These data clued that binding sites of A20 and NLRP3 with NEK7 might exist overlap, and A20 might competitively bind with NEK7 thus attenuating the activation of NEK7/NLRP3/pyroptosis axis.

### Disturbing NEK7 alleviates the progression of AKI

**Fig. 8:**
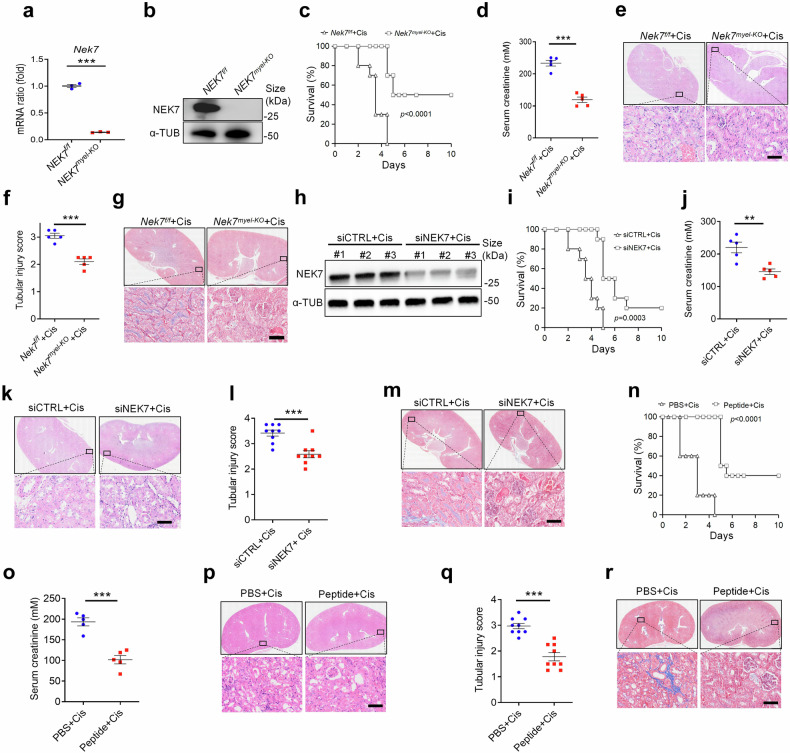
Targeting NEK7 can alleviate acute kidney injury. NEK7 knockout efficiency of myeloid cells from *Nek7*^*f/f*^ or *Nek7*^*myel-KO*^ mice were detected by quantitative PCR analysis (**a**) and immunoblotting (**b**). **c** Survival curves of the *Nek7*^*f/f*^ or *Nek7*^*myel-KO*^ mice that treated with Cis. (*n* = 10 per group). **d** Serum creatinine of mice as depicted in (**c**). (*n* = 5, mean ± SEM). ***P < 0.001. **e** Representative images of HE staining of the renal tissue sections. Scale bar, 50 μm. (n = 5). **f** Histological analysis of tubular injury of renal tissue sections as described in (**c**). (*n* = 5, mean ± SEM); ***P < 0.001. **g** Representative images of Masson staining of the renal tissue sections. Scale bar, 50 μm. (*n* = 5). **h** Immunoblots for NEK7 from renal tissue of Cis-treated mice that underwent in vivo NEK7 knockdown by siRNA. (*n* = 3) (# indicates the serial number of the individual mouse). **i** Survival of Cis-treated siCTRL mice or siNEK7 mice (*n* = 10). **j** Serum creatinine of mice as described in **i**. (*n* = 5, mean ± SEM). **P < 0.01. **k** Representative images of HE staining of renal tissue sections from Cis-treated siCTRL mice or siNEK7 mice. Scale bar, 50 μm, (*n* = 9). **l** Histological analysis of tubular injury of renal tissue sections as described in (**i**). (*n* = 9, mean ± SEM); ***P < 0.001. **m** Representative images of Masson staining of the renal tissue sections. Scale bar, 50 μm. (*n* = 5). **n** Survival curves of the mice that treated with Cis or Cis plus 12 h pretreated A20-derived-peptide(15 mg/kg). (*n* = 10 per group). **o** Serum creatinine of mice as described in (**n**) (*n* = 5, mean ± SEM). ***P < 0.001. **p** Representative images of HE staining of renal tissue sections from mice that were treated with Cis or Cis plus 12 h pretreated A20-derived-peptide. Scale bar, 50 μm, (*n* = 9). **q** Histological analysis of tubular injury of renal tissue sections as described in (**n**). (*n* = 9, mean ± SEM); ***P < 0.001. **r** Representative images of Masson staining of the renal tissue sections. Scale bar, 50 μm. (*n* = 5)

To confirm the effect of NEK7 on the progression of inflammation in AKI, NEK7 was specifically knocked-out in myeloid cells (*Nek7*^*myel-KO*^), and the knockout efficiency was verified (Fig. 8a, b). Subsequently, *Nek7*^*f/f*^ and *Nek7*^*myel-KO*^ mice were intraperitoneally injected with cisplatin. As expected, the survival time and their survival rate of *Nek7*^*myel-KO*^ mice have improved, and serum creatinine was less than *Nek7*^*f/f*^ after cisplatin induction (Fig. 8c, d). Histologically, cisplatin-induced tubular damage of *Nek7*^*myel-KO*^ mice was less severe, with reduced tubular injury and renal fibrosis (Fig. 8e-g). For further verification, NEK7 was knocked-down by siNEK7 delivered with a nanoparticle-based in vivo transfection reagent (Fig. 8h). The effect of siNEK7 exhibited a similar pattern to that observed in *Nek7*^*myel-KO*^ mice (Fig. 8i-m). Consistently, treatment with the A20-derived peptide also improved the survival of mice, along with alleviated the serum creatinine and renal injury of AKI mice (Fig. 8n-r). These data suggest that NEK7 is a promising target for AKI therapy, as it can attenuate ox-self-DNA-raised inflammation and improve the prognosis of AKI.

**Fig. 9:**
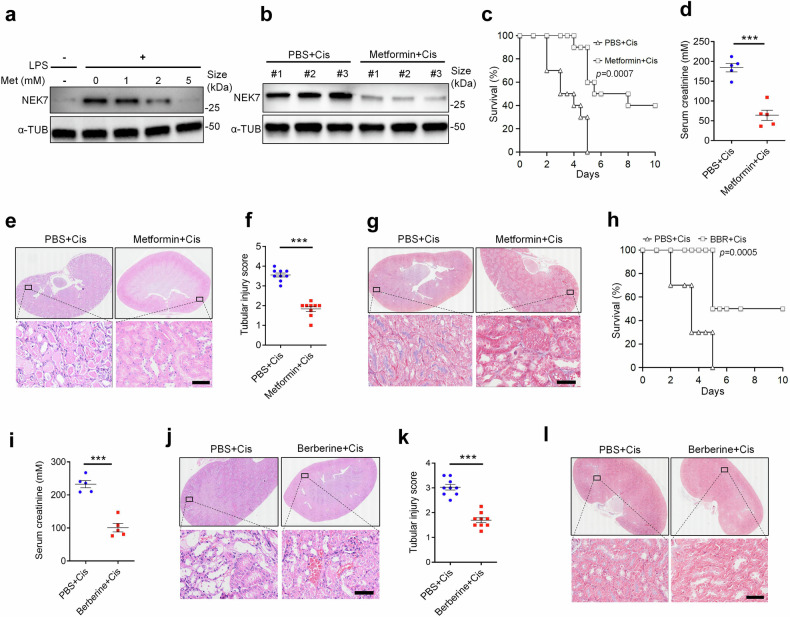
Medicines targeting NEK7 alleviates the progression of AKI. **a** Immunoblots for NEK7 from iBMDMs treated with LPS or LPS plus metformin for 0 mM, 1 mM, 2 mM, or 5 mM. **b** Immunoblots for NEK7 from renal tissue of mice treated with PBS plus Cis or metformin plus Cis (# indicates the serial number of individual mouse). **c** Survival of Cis-treated mice or Cis plus metformin-24 h-pretreated mice (*n* = 10 per group). **d** Serum creatinine of mice as described in (**c**). (*n* = 5, mean ± SEM). ***P < 0.001. **e** Representative images of HE staining of renal tissue sections from mice treated with PBS plus Cis or metformin plus Cis. Scale bar, 50 μm. (*n* = 9). **f** Histological analysis of tubular injury of renal tissue sections as described in (**c**). (*n* = 5, mean ± SEM); ***P < 0.001. **g** Representative images of Masson staining of the renal tissue sections. Scale bar, 50 μm. (*n* = 5). **h** Survival of mice treated with PBS plus LPS or berberine plus LPS. (*n* = 10 per group). **i** Serum creatinine of mice as described in (**h**). (*n* = 5, mean ± SEM). ***P < 0.001. **j** Representative images of HE staining of the renal tissue sections from mice as described in (**h**). Scale bar, 50 μm. (*n* = 9). **k** Histological analysis of tubular injury of renal tissue sections as described in (**h**) (*n* = 9, mean ± SEM); ***P < 0.001**. l** Representative images of masson staining of the renal tissue sections. Scale bar, 50 μm. (*n* = 5)

Furthermore, we investigated the potential therapeutic medicines targeting to NEK7 and evaluated their effects on alleviating AKI. A previous study discovered that low dose of metformin can limit the expression of NEK7 to alleviate inflammation.^42^ To verify this, we conducted immunoblotting, which confirmed that metformin downregulates NEK7 both in vitro (iBMDMs) and in vivo (Fig. 9a, b). The inhibition of NEK7 expression by metformin markedly ameliorated cisplatin-induced serum creatines, tubular injury, renal fibrosis, along with longer survival time and better survival rate (Fig. 9c–g). Additionally, we examined the effects of berberine, known to inhibit NEK7 activity in AKI mice.^43^ Consistently, berberine-treated mice demonstrated improved survival, decreased serum creatinine, attenuated renal injury, and decreased renal fibrosis compared with PBS-treated mice following injection of cisplatin (Fig. 9h-l). Thus, medicines that impact the activity of NEK7 and restrict NEK7 expression can mitigate the progression of AKI.

## Discussion

Our study highlights the crucial role of A20 in restricting self-DNA-induced NLRP3 inflammasome activation and pyroptosis, thereby limiting the progression of AKI. Increased release of oxidized self-DNA was detected in both human patients and AKI mouse model. Self-DNA ultimately activated the STING pathway and the NLRP3 inflammasome. Interestingly, targeting the NLRP3/pyroptosis axis, more effective than dampening the STING pathway, significantly improved the survival time and rate of AKI mice. A20 was screened out and identified as a self-limiting molecule with anti-inflammatory and anti-pyroptotic functions in vitro and in vivo. Notably, A20-derived peptide (P-II) generated by ourselves showed high affinity to NEK7 protein and demonstrated capability of interfering the binding of NEK7 to NLRP3 complex. Innovatively, we further investigated the crucial residues that support the binding of P-II and NEK7 by employing the AlphaFold3. By generating various point mutation of NEK7 and implementing BLI analysis and Co-IP experiment, we validated that the Lys140 of NEK7 is crucial for this binding. Furthermore, our data proved that Lys140 is also crucial for the binding of NEK7 and NLRP3 protein, mutation of which effect on the interaction of NEK7 and NLRP3 complex. These data clued that A20 might competitively bind with NEK7, thus dampening activation of NLRP3 inflammasome and attenuating pyroptosis. We also generated *Nek7*^*myel-KO*^ mice and employed pharmacological inhibitor of NEK7, such as metformin, berberine and A20-derived peptide, to explore the role of NEK7 in the development of AKI. Reduced protein levels and restrictive activity of NEK7 both attenuated NLRP3 inflammasome-mediated pyroptosis and ameliorated the survival time of AKI mice. Therefore, the A20-NEK7 axis is a promising therapeutic target for AKI.

Self-DNA originates from dying cells and is readily oxidized under conditions of oxidative stress and inflammation.^44^ Oxidatively modification of self-DNA confers resistance to exonuclease TREX1-mediated degradation and potentiates STING-dependent immune sensing, thereby amplifying the inflammatory response.^15,45^ Furthermore, activation of the STING pathway in patients and mouse models has been associated with intensified tubular inflammation and AKI progression.^37^ However, our current study reveals that deletion of STING only moderately improves the survival of AKI mice. Additionally, the therapeutic effect in *Sting*^−/−^ mice was less pronounced compared to DNase I-treated mice, suggesting that self-DNA may promote acute renal injury through other mechanisms.

Previous studies have indicated that ox-DNA can specifically bind to NLRP3, enabling the NLRP3 inflammasome complex. The strongly activated cGAS-STING axis resulting from DNA stimulation can further mediate the mobilization of the NLRP3 inflammasome, highlighting its crucial status in the NLRP3 inflammasome mediating ox-dsDNA-induced inflammation. We found that ox-self-DNA stimulation led to increased expression of NEK7 and NLRP3, as well as elevated cleavage of GSDMD. In addition, the NLRP3 inhibitor MCC950 significantly alleviated the progression of AKI. Thus, oxidized self-DNA activates the NLRP3 inflammasome and promotes the development of AKI. Notably, utilizing DNase I and the GSDMD inhibitor disulfiram was more effective in alleviating AKI than the NLRP3 inhibitor MCC950 in our study, which might be because only a part of the self-DNA was oxidized. Non-oxidized DNA can be sensed by AIM2, the activation of which also results in pyroptosis and secretion of IL-1β and IL-18.^6^

We observed markedly increased expression of A20 after oxidized self-DNA stimulation. A20 was previously reported to serve as crucial negative feedback molecular in inflammatory processes, capable of maintaining the homeostasis of cells and organs by alleviating cell death, such as apoptosis and necroptosis.^40,46^ In our study, multiple signaling pathways, including the NOD-like receptor (NLR), TNF-α, and IL-17 signaling pathways, were upregulated after ox-DNA stimulation, and these pathways are known to further activate the downstream NF-κB signaling pathway.^47,48^ A20 is an immediate transcriptional target of NF-κB and plays a pivotal role in the negative feedback control of the NF-κB signaling pathway.^49^ Our data demonstrated that the increased expression of A20 under ox-DNA stimulation was abrogated when the NF-κB signaling pathway was inhibited by BAY11-7082. Thus, A20 may act as a self-limiting molecule in reacting to ox-DNA stimulation, protecting the kidney from inflammation and tissue damage.

Consistent to previous study, our result indicated a role of A20 in negatively regulating STING pathway and NLRP3 inflammasome. Moreover, our published data have revealed that A20 possessed the capability of attenuating NLRP3 inflammasome via impacting NEK7.^29^ Although the study has suggested the ubiquitinated residues which is crucial for the ubiquitination of NEK7, the sites supported the binding of NEK7 and A20 has not been elucidated. In this study, we further investigated the crucial residues that support the binding of P-II and NEK7, and we validated that the Lys140 of NEK7 is crucial for this binding. mutation of Lys140 also decreased the binding between A20 and NEK7 protein. Interestingly, Lys40 is located at the C-terminus lobe of NEK7, and our previous studies have demonstrated that the C-terminus lobe of NEK7 is crucial for its binding with A20. Therefore, the results of this study are consistent with our earlier findings. Furthermore, these mutants also interfered the interaction of NEK7 and NLRP3 complex. Therefore, A20 is capable of attenuating the binding of NEK7 to NLRP3 complex, thus dampening ox-self-DNA-induced pyroptosis.

Furthermore, we verified the capability of A20 and P-II in vivo. Overexpression of A20 could improve the survival of AKI mice and mitigate their kidney injury. However, treatment efficiency varies across different pharmaceuticals. Administration of Anc80L65 alone only moderately improved the survival rate of AKI mice, while TNF-α was more efficient in alleviating AKI. This discrepancy may be due to Anc80L65 specifically targeting the kidney mesenchymal cell lineage, without affecting the macrophages recruited to the kidney during the progression of AKI.^50^ Altogether, A20 is potential therapeutic target for attenuating cisplatin-induced AKI through its regulation of the self-DNA-mediated STING pathway and NLRP3-driven pyroptosis.

In summary, A20 attenuates self-DNA-mediated inflammation in AKI by dampening NEK7/NLRP3/pyroptosis signaling pathway in AKI. Both NEK7 and A20 are promising targets, with their downregulation or upregulation through genetic techniques or small molecules representing potential therapeutic strategies for AKI.

## Materials and methods

### Mice

The A20 floxed (*A20*^*flox/flox*^) mice, which have two LoxP sites flanking exon III of the *tnfaip3* gene, were established at Cyagen (Shanghai, China). Using the Cre/LoxP system, *A20*^*flox/flox*^ mice were crossed with LysM-Cre transgenic mice to generate myeloid-specific A20 conditional knockout mice (*A20*^*myel-KO*^). *Sting*^*−/−*^ mice were purchased from Jackson Laboratory. The *Nek7* floxed (*Nek7*^*flox/flox*^) conditional knockout mice were also established at Cyagen (Shanghai, China). *Nek7*^*flox/flox*^ mice were crossed with LysM-Cre transgenic mice to generate myeloid-specific Nek7 knockout mice (*Nek7*^*myel-KO*^). Reproduction of these mice follows Mendelian genetic laws, and after birth, the appearance, development, morphology of the kidneys, and the shape of the renal tubules were also determined (Supplementary Fig. 6). The results indicate no obvious disparity between *Nek7*^*myel-KO*^ mice and its littermates. C57BL/6 J female and male mice, aged 6–8 weeks, were purchased from Vital River Laboratories (VRL) in Beijing and housed in a specific pathogen-free (SPF) animal facility. The breeding conditions for mice include a temperature range of 20–26 °C, humidity levels between 40 and 70%, and ammonia concentrations below 20 ppm. All animals were handled in accordance with national guidelines for the housing and care of laboratory animals, as well as the regulations of the Animal Ethics Committee of Sichuan University. This study was approved by the Ethics Committee of West China Hospital of Stomatology of Sichuan University (Ethical code: WCHSIRB-D-2023-260).

### Cell line

Mouse fibroblasts (L929) and human embryonic kidney cells (HEK293T) were purchased from the American Type Culture Collection (ATCC, USA). The immortalized murine bone marrow-derived macrophage (iBMDM) cell line was generously provided by Dr. Feng Shao from the National Institute of Biological Sciences, Beijing. Primary BMDMs and iBMDMs were cultivated in Dulbecco’s modified Eagle’s medium (Gibco, Lot#8122093), while L929 cells were cultured in Roswell Park Memorial Institute medium (Gibco, Lot#11875119). All media were supplemented with 10% fetal bovine serum (Gibco, Cat#26140095), 50 U/mL penicillin, and 50 mg/mL streptomycin (Cytiva, Cat#SV30010). PlasmocinTM Prophylactic (5 μg/mL, InvivoGen, Cat#ant-mpp) was added to the culture medium to prevent mycoplasma contamination. Cells were cultured at 37 °C with 5% CO_2_. All cell lines were quality-controlled by monitoring their morphological features and functionalities.

### Antibodies

The specific information of antibodies is shown in Supplementary Table 1.

### Human Samples

Human serum samples were obtained from the blood of active sepsis patients (*n* = 9) and healthy donors (*n* = 9). This study was approved by the Ethics Committee of West China Hospital of Stomatology of Sichuan University (Ethical code: WCHSIRB-D-2022-107).

### Peptide

A20-derived peptide was constructed by GeneWiz company. The sequence of peptide (P-II): KLVALKTNGDGNCLMHAACQYMWGVQDTD. The sequence of control peptide: CCKLTAKNGRSYEYGTEIFKNRFGPKCVFTEC.

### Primary BMDMs isolation

Primary bone marrow-derived macrophages (BMDMs) were isolated from 6- to 8-week-old C57BL/6J male mice. The mice were sacrificed and soaked in 75% alcohol for 1–2 min. The femurs and tibias were removed and immersed in PBS containing 500 U/mL penicillin and 500 mg/mL streptomycin for 3 min. The marrow cavities were repeatedly rinsed with DMEM using a sterile 1 mL syringe. The cell suspension was collected and centrifuged at 300 × *g* for 10 min, and the supernatant was discarded. The cell pellet was resuspended in 5 mL red cell lysis buffer (Bio Sharp, Cat#BL503A) on ice for 5 min, followed by centrifugation, and the supernatant was removed. The cells were then resuspended in DMEM complete culture medium supplemented with 10% FBS and 30% L929 cell-conditioned medium as an induction factor.^51^ Half of the medium was changed every other day for 5–7 days. The suspended cells were removed, and the adherent BMDMs were digested with trypsin and plated on a six-well plate at a density of 1 × 10^6^ cells/well. The incubation of primary BMDMs followed the same conditions as described for drug administration when assays under stimulating conditions were needed.

### Cell treatment

Cells were plated in six-well plates at a density of 1 × 10^6^ cells/well and cultured until a majority of cells were attached to the bottom. To stimulate the NLRP3 inflammasome pathway, LPS (Sigma, E. coli O111:B4, Cat#L2630) was added (2 μg/mL for HK-2 or HEK293T and 100 ng/mL for BMDMs and iBMDMs), and 2.5 mg/mL ATP (Roche, Lot#40666727) was added 4 h later for another 1 h before sample collection. For A20-derived peptide, peptide was dissolved in ddH_2_O to 1 mM and pretreated to cells in a dose of 0, 5, 10, 20 µM for 12 h before LPS.

### Immunoblotting

For immunoblot analysis of kidney tissue, total protein lysates were prepared from renal tissue, which was ground in liquid nitrogen and then added to lysis buffer in a Total Protein Extraction Kit (Signalway Antibody, Cat#PE001). Samples were centrifuged at 12,000 rpm for 15 min at 4 °C, and the supernatant was collected immediately. For immunoblot analysis of cells, cultured cells after treatment were lysed with lysis buffer, which was premixed with protease and phosphatase inhibitors (Signalway Antibody, Cat#PE001). The samples were centrifuged at 12,000 r/min for 15 min at 4 °C, and the supernatant was collected immediately. Ultimately, both types of protein samples were then mixed with 4× Laemmli loading buffer (Bio-Rad, Cat#1610747) and denatured by boiling at 95 °C for 5 min.

Prepared protein samples were subjected to 6-15% SDS polyacrylamide gels and separated according to the protein concentrations. Then, the protein bands were transferred to polyvinylidene difluoride (PVDF) membranes by a wet transfer apparatus (Bio-Rad, USA). Then, membranes containing protein were blocked with Tris-buffered saline with Tween (TBST) containing 5% bovine serum albumin (BSA, Biofroxx, Cat#4240GR500) for 2 h and then incubated overnight at 4 °C with primary antibodies (shown in Antibodies), followed by incubation with a horseradish peroxidase-conjugated IgG (Signaling Antibody, Goat anti-Rabbit Cat#L3012, Goat anti-Mouse Cat#L3032) for 1 h. The protein bands were detected with an enhanced chemiluminescence kit (Millipore, Cat#WBKLS0500) using a gel imaging system (Bio-Rad, USA) for visualization and standardization of the amount of protein loaded. α-Tubulin was used as the internal control.

### Coimmunoprecipitation

Cells were lysed in mild lysis buffer with a protease inhibitor cocktail (Cell Signaling Technology, Cat#5872S) on ice for 10 min to perform the coimmunoprecipitation (co-IP) assay. Samples were then centrifuged at 12,000 rpm at 4 °C for 10 min, and the supernatant was incubated with the corresponding primary antibody (anti-HA, 1:100, Cell Signaling Technology, Cat#3724S) overnight at 4 °C. On the next day, magnetic beads were added and incubated for 1 h. The immunocoprecipitated protein was washed three times by MLB and then heated with 4× Laemmli loading buffer at 100 °C for 10 min. Samples were then subjected to western blot analysis.

For biotin mediated immunoprecipitation, plasmids of HA-NEK7 (WT, K130A, L132R, K140A and Tri-MUT) were transfected into HEK293T cells for 2 days. Then, cells were lysed in MLB with inhibitor cocktail for 10 min at 4 °C. Cell lysates were collected and centrifuged at 12,000 rpm for 15 min at 4 °C and the supernatant was incubated with biotin conjunction peptide overnight at 4 °C. On the next day, streptavidin magnetic beads(Invitrogen, Lot#91221939) were added and incubated for 1 h. The immunocoprecipitated protein was then subjected to washing, heating, and western blot analysis.

For BLI analysis, 3×HA-WT-NEK7 or 3×HA-Tri-MUT-NEK7 plasmids were transfected into HEK293T cells. Post-transfection, proteins were purified with HA antibody via coimmunoprecipitation and then eluted with 0.1 M glycine, Ph2.0. Then, samples were neutralized with 3 M NaOH and subjected to BLI analysis.

### Drug administration

Aristolochic acid I sodium salt was dissolved in PBS to 1 mg/mL and injected intraperitoneally at a dose of 10 mg/kg (body weight) every 24 h for 3 days; cisplatin was dissolved in PBS to 1.5 mg/mL for one-time intraperitoneal 25 mg/kg injection; LPS (Sigma, Cat#L2630) was dissolved in PBS to 2.5 mg/mL for one-time intraperitoneal 25 mg/kg injection; deoxyribonuclease I (DNase I, Sigma, Cat#SLBV1446) was dissolved in PBS to 1 mg/mL and injected intraperitoneally at a dose of 10 mg/kg at 4 h before cisplatin or AA I injection and every 24 h after injection. Disulfiram (MedChemExpress, CAS:97-77-8) was diluted in corn oil to 12.5 mg/mL and injected 50 mg/kg intraperitoneally every 24 h after cisplatin or AA I administration. Cu(II) (Sigma, Cat#344419) was dissolved in PBS to 30 µg/mL and administered intraperitoneally 6 h before disulfiram at a dose of 0.15 mg/kg; TNF-α (Peprotech, Cat#315-01 A) was dissolved in PBS to 10 µg/mL and injected at 100 µg/kg once through the caudal vein 24 h before cisplatin injection. Metformin (Sigma, Cat#D150959) was dissolved in PBS to 5 mg/mL and injected intraperitoneally at a dose of 50 mg/kg 24 h before cisplatin or AA I injection and every 24 h after injection. MCC950 (MedChem Express, CAS:256373-96-3) was dissolved in PBS to 5 mg/mL and injected intraperitoneally at a dose of 50 mg/kg 12 h before cisplatin or AA I injection and every 12 h after injection; berberine (APExBIO, Cat#N1368) was dissolved in PBS to 5 mg/mL and 5 mg/kg injected intraperitoneally at 36, 24 and 12 h before cisplatin; A20-derived-peptide was dissolved in ddH_2_O to 3 mg/mL and injected intraperitoneally at a dose of 15 mg/kg 1 h before cisplatin. To supervise the effects of various treatments on survival time and rate, the mice were observed, and survival curves were generated. To investigate the specific mechanisms, whole blood was retrieved from the orbit, serum was separated through centrifugation on the 4th day, and the mice were then sacrificed by cervical dislocation. Mice were randomly assigned in experiments, while there was no blinding during the animal experiments.

### Bulk RNA-seq

BMDMs were cultured in six-well plates at a density of 1 × 10^6^ cells/well and treated with 5 µg ox-dsDNA90 or CDNs. After 6 h, cells were lysed with 1 mL buffer RZ (TIANGEN, Cat#DP419) and sent to Novogene for bulk RNA-seq. Genes were considered significantly differentially expressed if the *P* value < 0.05 and they showed a ≥ 2.0-fold change. GSEA was performed with GSEA software (http://www.broad.mit.edu/GSEA). A heatmap was generated with Morpheus.

### Quantitative real-time PCR (qRT‒PCR)

Total RNA was extracted from around 1 × 10^6^ cultured cells per population and purified by employing an RNA simple Total RNA Kit (TIANGEN, Cat#DP419), Following column purification, RNA was immediately reversed to cDNA by implementing the PrimeScript reverse transcription reagent kit (Takara, RR047A) according to the manufacturer’s instructions. The concentration of RNA was measured with a NanoDrop 2000 (Thermo Fisher Scientific, USA). Reverse transcription polymerase chain reaction (qRT‒PCR) was performed using SYBR Premix Ex Taq (Takara, CAT#172-5124) in an ABI7500 real-time PCR system (Applied Biosystems, CA). The primer sequences are shown in Supplementary Table 2. Relative gene expression was presented as fold changes normalized by *Gapdh* using the 2^−ΔΔCt^ method.

### ELISA

To measure the levels of CXCL10, IFN-β, IL-1β, and IL-18, mouse serum or cell culture medium was collected and measured by ELISA (NeoBioscience, China) according to the manufacturer’s instructions.

### LDH release

To measure the release of LDH, cell culture medium was collected and measured by CytoTox96@Non-Radio cytotoxicity Assay (Promega, REF#G1781) according to the manufacturer’s instructions.

### Self-DNA isolation and transfection

Self-DNA was isolated from the mouse kidney tissue using a DNeasy®Blood & Tissue Kit (QIAGEN, REF#69504) according to the manufacturer’s instructions. The isolated self-DNA were mixed with PicoGreen reagent, and then detected via employing SpectraMax iD3 microplate reader (Molecular Devices), with 502 nm incident ray and 523 nm emergent light. Consequently, DNA quantification was measured by comparing with standard curve. Cells were seeded in 6-well plates at a density of 1 × 10^6^/well and transfected with 5 µg DNA using Lipofectamine 3000 (Thermo Fisher Scientific, Cat#L3000015) according to the instructions. The 8OH-dG level in samples was detected by a DNA Damage 8OH-dG ELISA Kit (StressMarq Biosciences, CAT#SKT-120). Oxidized dsDNA90 is used as a positive control. To produce ox-dsDNA90, synthesized normal DNA was irradiated with 70 Gy by X-ray irradiation (Rad source: Rs2000Biological Irradiator).

### siRNA-mediated gene knockdown

NEK7 in cells was knocked down by mouse siRNA (GenePharma). Cells were transfected with Lipofectamine RNAimax (Invitrogen, Cat#13778030) according to the manufacturer’s instructions. In vivo, gene knockdown was performed by Nanoparticle-based in Vivo Transfection Reagent (Millipore, Cat#5031) according to the manufacturer’s instructions. After 48 hours of transfection, qRT‒PCR or immunoblot analysis was performed to test knockdown efficiency. siRNA of NEK7 consists of pools with three target-specific 19- to 25-nucleotide siRNAs.

### Lentivirus-mediated overexpression

iBMDMs were infected with lentivirus encoding green fluorescent protein (GFP) or mouse A20/GFP purchased from GeneChem (Shanghai, China). Cells were infected with lentivirus at MOI = 10. 48 h after injection, positive cells were selected by puromycin (Invitrogen, Cat#A1113803) and used in subsequent experiments.

### Plasmid-mediated gene expression

Mouse HA-NEK7, FlAG-A20, and NLRP3 cDNA were amplified by employing standard PCR techniques from a mouse cDNA library. Subsequently, diverse cDNAs were inserted into mammalian expression vectors pcDNA3.1(+), pRK5, or pCMV6 with 3×Flag or 3×HA tags. For transient transfections, Lipofectamine 3000 was utilized according to the manufacturer’s instructions.

### The predicted structure and interaction of NEK7 and peptide

The structure of mouse NEK7 protein and peptide P-II (sequence: KLVALKTNGDGNCLMHAACQYMWGVQDTD) complex were predicted by AlphaFold3 (https://www.nature.com/articles/s41586-024-07487-w). Figures were prepared using Chimera v1.16 (https://www.cgl.ucsf.edu/chimera/docs/credits.html).

### Mass spectrometry

Primary BMDMs were cultured to 90% density in 10 cm dishes and then treated with 30 µg ox-dsDNA90 (General Biol, 70 Gy treated) using Lipofectamine 3000 for 6 h. Then, samples were lysed in a mild lysis buffer plus protease inhibitor cocktail on ice for 10 min and centrifuged at 12,000 rpm at 4 °C for 10 min. Immunoprecipitation was performed with A20 antibody (1:100, Santa Cruz, Cat#sc-166692) and Pierce Protein A/G Magnetic Beads. The protein complex on magnetic beads was sent to Shanghai Luming Biological Technology Co., Ltd. (Shanghai, China) and subjected to mass spectrometry for peptide sequencing and data analysis.

### Bio-Layer Interferometry (BLI) analysis

The peptide (Bootech BioScience &Technology) was immobilized separately on Octet® AR2G Biosensors (Sartorius, Lot#2308017311) at a concentration of 400 μg/ml for 500 s. Association and dissociation curves were obtained through the addition of a dilution series of recombinant human NEK7 protein (SinoBiological, CAT#11534-H20B) (39.0625 to 1250 nM) for 120 s followed by dissociation for 120 s. Interference patterns for association or dissociation were measured in real-time to generate a response profile on the Octet® System Octet 96 Red instrument (Sartorius). A 1:1 binding model was used to evaluate the Kinetic parameter.

### Renal function and histological analysis

Renal function was evaluated based on serum creatinine level and histological features. Serum creatinine was determined in the Clinical Pharmacology Lab, Clinical Trial Center, WCH, SCU. The kidneys were extracted from euthanized mice, immediately fixed with 4% paraformaldehyde, and embedded in paraffin. Furthermore, the samples were cut into 5 µm thick sections. These tissue samples were subjected to hematoxylin-eosin (HE) and periodate according to standard protocols with HE reagent (Biosharp, Cat#BL702B, BL703B) and Masson reagent (Servicebio, Cat#G1006-100ML). The pathological changes were detected through optical microscopy, and the tubulointerstitial injury score was determined as previously reported.

### Immunofluorescence staining

For preparation, BMDMs were seeded on coverslips in 12-well plates. 4% paraformaldehyde was employed on fixed cells or frozen tissue slides. Then, cells or tissue were permeabilized with 0.2% or 0.5% Triton X-100 in PBS for 15 min at room temperature. Tissue slides were blocked for 90 min with 10% goat serum (Gibco) in PBS and washed 3 times with PBS. Cells were blocked in PBS with 2% goat serum for 30 min at room temperature and washed 3 times with PBS. Thereafter, cells were incubated overnight at 4 °C in primary antibodies (anti-F4/80 antibody, 1:100, Abcam, Cat#ab6640; anti-DNA/RNA damage antibody 8OH-dG, 1:100, StressMarq Biosciences, Cat#SMC-155C; anti-NEK7,1:200, Abcam, Cat#ab133514; anti-A20,1:100, CST, Cat#5630S) diluted in 1% BSA. After three washes with PBS, secondary antibodies (Fluorescein (FITC)-conjugated AffiniPure Goat anti-Rabbit IgG, Lot#143113; Alexa Fluor®647-conjugated AffiniPure Goat anti-Mouse IgG, Lot#141733. Life Technologies, 1:500) were diluted in 1% BSA and incubated for 1 h. Tissue slides were incubated overnight at 4 °C in primary antibodies (anti-DNA/RNA damage antibody, 1:100, StressMarq Biosciences, Cat#SMC-155C). Thereafter, cells and tissue slides were both incubated with DAPI (Life Technologies, 1:500, Cat#62248) for 10 min. Olympus FV1000 confocal microscopy was employed to capture images.

### Anc80L65 AAV vector

The Anc80L65 AAV vector was produced by triple plasmid transfection of HEK293 cells as previously described.^52^ HEK293 cells were segmented into 10-layer cell stacks containing 1 liter of DMEM complete culture medium supplemented with 10% FBS 4 days before transfection, and transfection was carried out after replacing the culture medium with serum-free medium. PEI-based transfections were implemented on 10-layer cell stacks of 75–90% confluent monolayers of HEK293 cells, maintaining the PEI/DNA max ratio at 2:1 (w/w). Then, cells were supplemented with adequate SFM 48 h after transfection, and the virus was harvested and purified by iodixanol gradient centrifugation 72 h after continuing culture. The genome titer (GC/mL) of the Anc80L65 AAV vector was determined by digital droplet polymerase chain reaction (ddPCR) using the forward primer 5′-GCAGACATGATAAGATACATTGATGAGTT-3′, reverse primer 5′- AGCAATAGCATCACAAATTTCACAA-3′, and probe 5′-Fam- AGCATTTTTTTCACTGCATTCTAGTTGTGGTTTGTC-BHQ-3’. All vectors used in this study passed the endotoxin assay using the end-point chromogenic endotoxin test kit (Xiamen Bioendo Technology, Cat#ECV1250V).

### Statistical analyses

All of the data reported in this study were analyzed by SPSS 23.0 software or GraphPad Prism 5.0. Data displaying a normal distribution are presented as the mean ± standard error of the mean (SEM). Statistically significant differences between the two groups were evaluated by employing Student’s *t* test. For comparisons of more than two groups, one-way analysis of variance with the least significant difference followed by Tukey’s post hoc test was utilized. *P* values and group sizes are presented in the corresponding figures and legends. *P* < 0.05 was considered statistically significant.

## Supplementary information

Supplementary Materials

## Data Availability

The RNA-seq data reported in this paper have been deposited in the Gene Expression Omnibus (GEO) database, https://www.ncbi.nlm.nih.gov/geo (accession nos. GSE219187, GSE219188). All of the other study data are included in the article and/or Supporting Information.
