# Supplementary material for: A20 attenuates oxidized self-DNA-mediated inflammation in acute kidney injury
Source: Signal Transduct Target Ther. 2025 Apr 25;10:154. doi: 10.1038/s41392-025-02194-y (PMC12032302; doi:10.1038/s41392-025-02194-y)
Supplement: Supplementary file 1 — Supplementary Materials [file 41392_2025_2194_MOESM1_ESM.docx]

Supplementary Materials for

**A20 attenuates oxidized self-DNA-mediated inflammation in acute kidney injury**

Hanwen Li, Yongyao Wu, Lisha Xiang, Qing Zhao, Lu Liu, Zhixiong Zhu, Weimin Lin, Zhan Li, Yang Yang, Yiting Ze, Lulu Zhang, Ping Fu, Yingqiang Guo, PingZhang, Bin Shao.

Correspondence to: drguoyq@wchscu.cn; pingzhang68@hotmail.com; sklbshaobin@scu.edu.cn;

**This PDF file includes:**

Figures. S1 to S6

Tables. S1 to S2

Figure. S1.
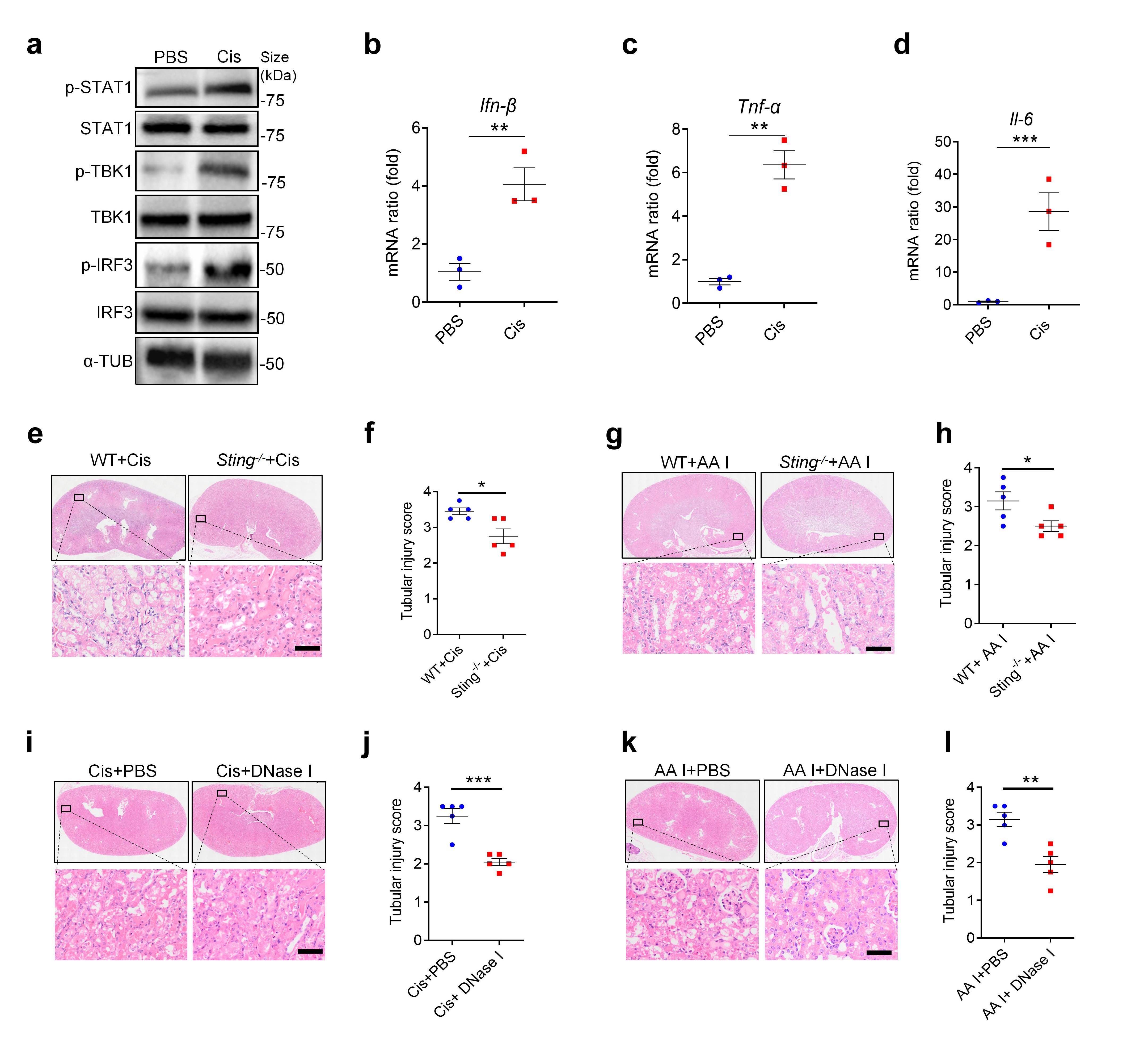


**Ox-self-DNA promotes the progression of AKI by activating STING signaling pathway. a,** Immunoblots for STAT1 and phosphorylated STAT1, TBK1 and phosphorylated TBK1, IRF3 and phosphorylated IRF3 of renal tissue from mice with PBS or Cis treatment. **b, c, d**, Quantitative PCR analysis of *Ifn-β* (b), *Tnfα* (c) and *Il-6* (d) mRNA in renal tissue of mice treated with PBS or Cis. The expression of *Gapdh* mRNA was used to normalize the results. (n = 3, mean ± SEM); ***P* < 0.01, ****P* < 0.001. **e**, Representative images of HE staining of the renal tissue sections from WT mice and *Sting^-/-^* mice intraperitoneally injected with Cis. Scale bar, 50μm. (n = 5). **f,** Histological analysis of tubular injury of renal tissue sections as described in **e**. (n = 5, mean ± SEM); **P* < 0.05. **g**, Representative images of HE staining of the renal tissue sections from WT mice and *Sting^-/-^* mice intraperitoneally injected with AA I. Scale bar, 50μm. (n = 5). **h**, Histological analysis of tubular injury of renal tissue sections as described in **g**. (n = 5, mean ± SEM); **P* < 0.05. **i**, Representative images of HE staining of the renal tissue sections from Cis plus PBS or Cis plus DNase I treated mice. Scale bar, 50μm. (n = 5). **j**, Histological analysis of tubular injury of renal tissue sections as described in **i**. (n = 5, mean ± SEM); ****P* < 0.001. **k**, Representative images of HE staining of the renal tissue sections from AA I plus PBS or AA I plus DNase I treated mice. Scale bar, 50μm. (n = 5). **l**, Histological analysis of tubular injury of renal tissue sections as described in **k**. (n = 5, mean ± SEM); ***P* < 0.01.

Figure. S2.
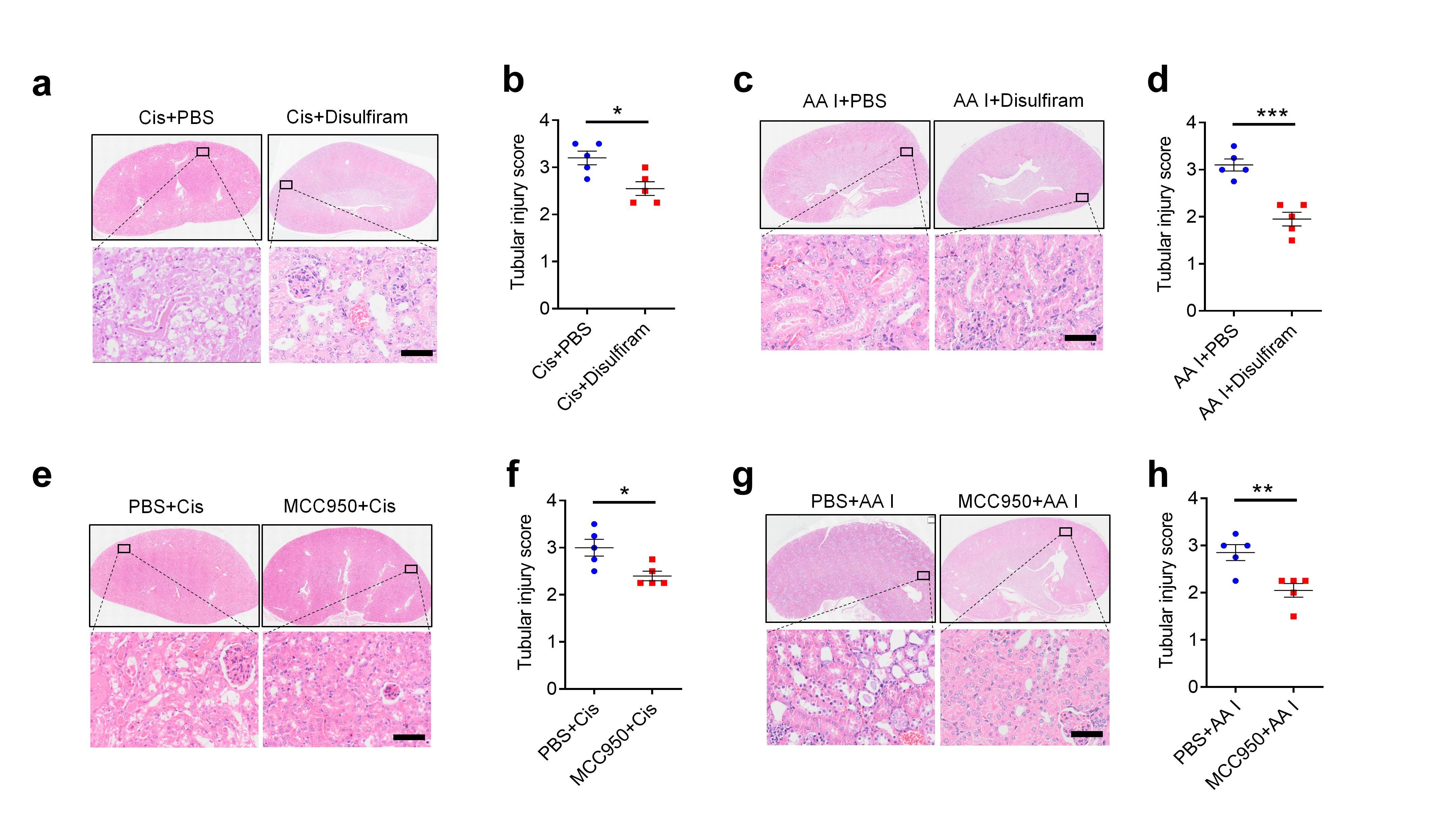


**Ox-self-DNA promotes the progression of AKI by facilitating pyroptosis. a**, Representative images of HE staining of the renal tissue sections from mice injected with Cis plus PBS or Cis plus disulfiram. Scale bar, 50μm. (n = 5). **b,** Histological analysis of tubular injury of renal tissue sections as described in **a**. (n = 5, mean ± SEM); **P* < 0.05. **c**, Representative images of HE staining of the renal tissue sections from mice injected with AA I plus PBS or AA I plus disulfiram. Scale bar, 50μm. (n = 5). **d**, Histological analysis of tubular injury of renal tissue sections as described in **c**. (n = 5, mean ± SEM); ****P* < 0.001. **e**, Representative images of HE staining of the renal tissue sections from mice injected with Cis plus PBS or Cis plus MCC950. Scale bar, 50μm. (n = 5). **f**, Histological analysis of tubular injury of renal tissue sections as described in **e**. (n = 5, mean ± SEM); **P* < 0.05. **g**, Representative images of HE staining of the renal tissue sections from mice injected with AA I plus PBS or AA I plus MCC950. Scale bar, 50μm. (n = 5). **h**, Histological analysis of tubular injury of renal tissue sections as described in **g**. (n = 5, mean ± SEM); ***P* < 0.01.

Figure. S3.


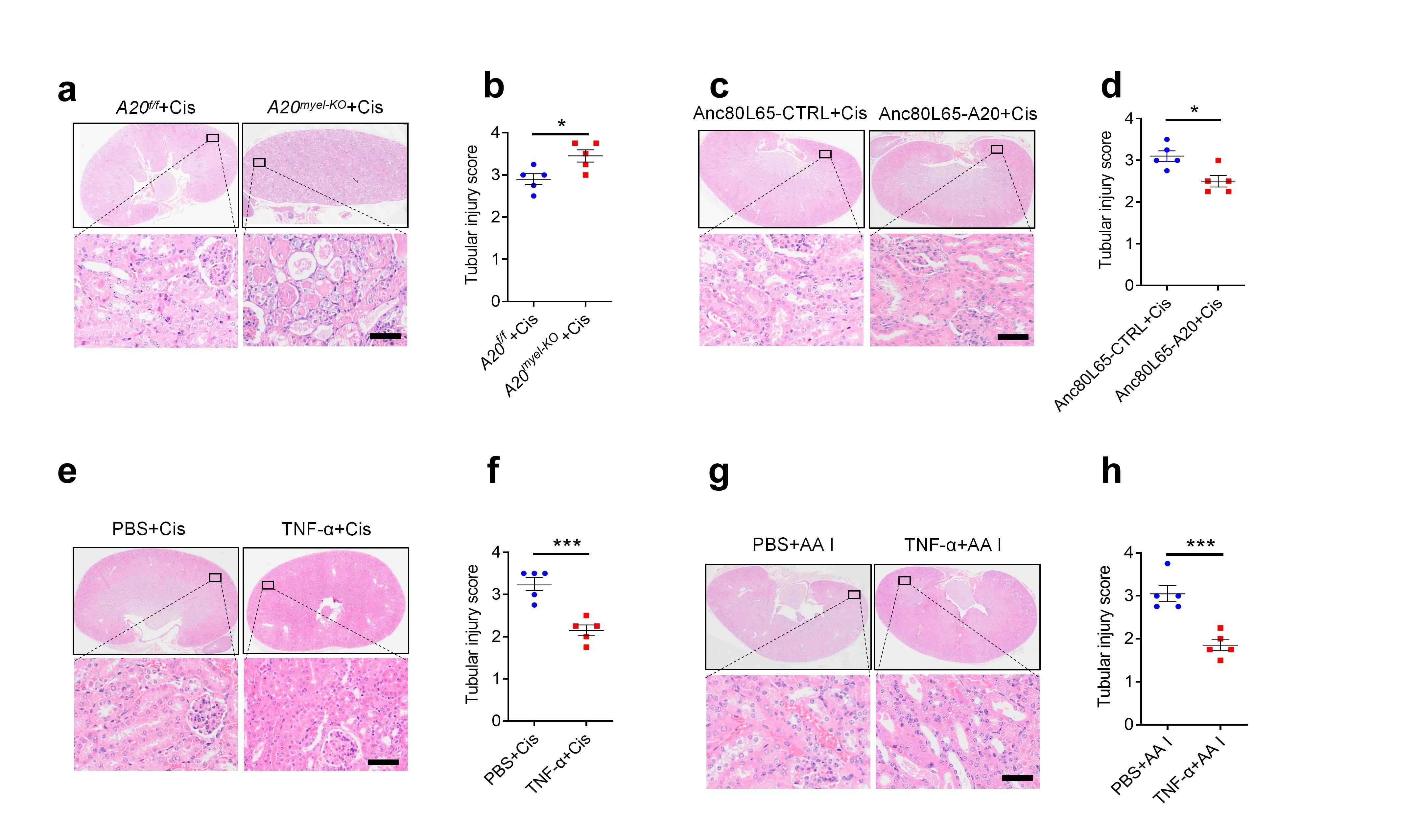


**A20 inhibits ox-DNA-induced pyroptosis and limits the progression of AKI. a**, Representative images of HE staining of the renal tissue sections from Cis treated *A20^f/f^* or *A20^myel-KO^* mice. Scale bar, 50μm. (n = 5). **b,** Histological analysis of tubular injury of renal tissue sections as described in **a**. (n = 5, mean ± SEM); **P* < 0.05. **c**, Representative images of HE staining of the renal tissue sections from mice treated with Anc80L65-CTRL plus Cis or Anc80L65-A20 plus Cis. Scale bar, 50μm. (n = 5). **d**, Histological analysis of tubular injury of renal tissue sections as described in **c**. (n = 5, mean ± SEM); **P* < 0.05. **e**, Representative images of HE staining of the renal tissue sections from mice injected with Cis plus PBS or Cis plus TNF-α. Scale bar, 50μm. (n = 5). **f**, Histological analysis of tubular injury of renal tissue sections as described in **e**. (n = 5, mean ± SEM); ****P* < 0.001. **g**, Representative images of HE staining of the renal tissue sections from mice injected with AA I plus PBS or AA I plus TNF-α. Scale bar, 50μm. (n = 5). **h**, Histological analysis of tubular injury of renal tissue sections as described in **g**. (n = 5, mean ± SEM); ****P* < 0.001.

Figure. S4.

**
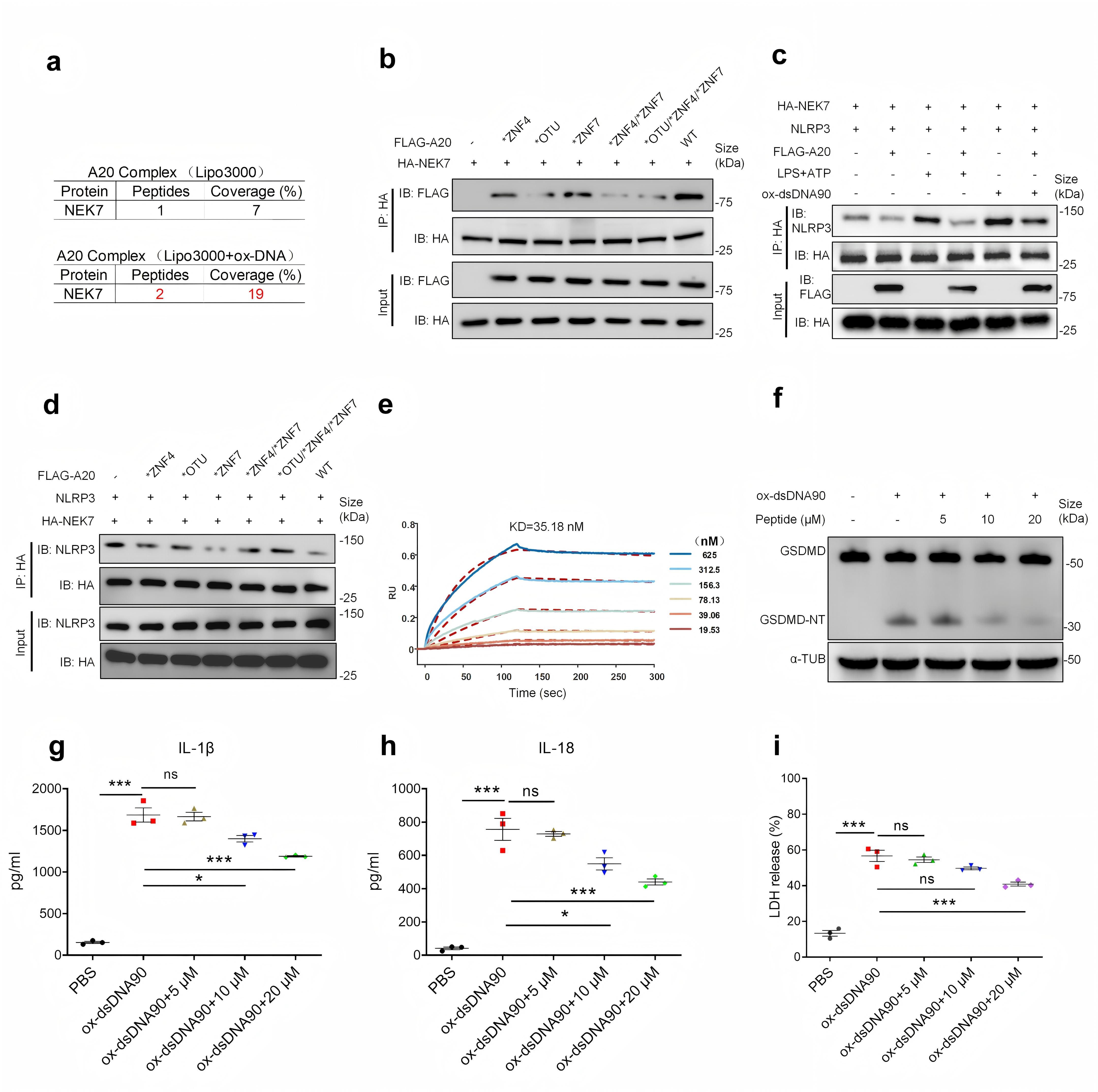
**

**A20 binds with NEK7 and impede the interaction of NEK7 and NLRP3. a,** BMDMs were treated with or without ox-dsDNA90, then A20 was immunoprecipitated with M2 beads and the binding proteins were analyzed by mass spectrometry. **b,** HEK293T were transiently transfected with indicated A20 associated/derived plasmids, including individual mutation of OTU, ZnF4 or ZnF7 domains (*OTU, *ZnF4, *ZnF7), simultaneous mutation of ZnF4 and ZnF7 (*ZnF4/*ZnF7) and combined mutation of OTU, ZnF4 and ZnF7 (*OTU/*ZnF4/*ZnF7). Protein level of A20 in immunoprecipitates of NEK7 was detected by immunoblotting. **c,** NLRP3 and HA-NEK7 plasmids were transiently transfected into HEK293T cells with or without transient transfection of FLAG-A20. Cells were stimulated with PBS, LPS plus ATP or ox-dsDNA90. Lysates were immunoprecipitated with an anti-HA antibody and the level of specific proteins were detect by immunoblot. **d,** HA-NEK7, NLRP3, and indicated plasmids of FLAG-A20 were transiently transfected into HEK293T. Immunoprecipitates were immunoblotted with the indicated antibodies. **e,** The kinetic interaction of recombinant human NEK7 protein and pepitide P-II were detected by BLI analysis. Fitting curve was shown as dotted line. **f,** Immunoblots for total and cleaved GSDMD from BMDMs treated by ox-dsDNA90 with 12 h pretreated P-II (0, 5, 10, 20μM). **g, h, i,** IL-1β (g) and IL-18 (h) level of supernatant from BMDMs culture medium as indicated in **f**, with cell death determined by LDH release (i) (n = 3, mean ± SEM). **P* < 0.05, ****P* < 0.001.

Figure. S5.

**
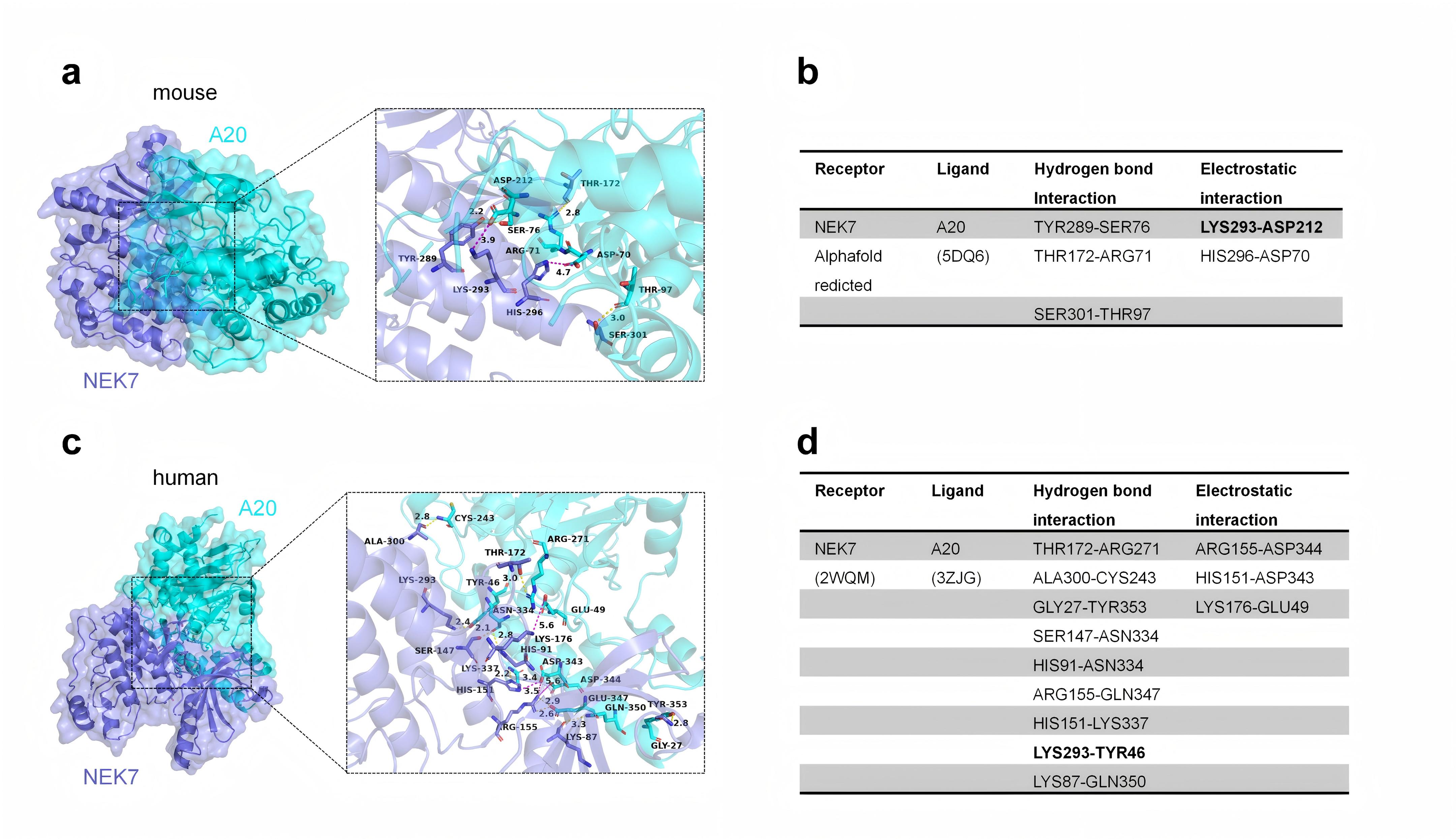
**

**Potential specific sites of A20 and NEK7 both in mouse and human. a-d,** The predicted protein structure of NEK7 (blue) and A20 (mint green) of mouse(a) or human(c). Dashed lines indicate predicated hydrogen bonds (orchid) and electronstatic interaction (yellow) of mouse (b) or human(d).

Figure. S6.

**
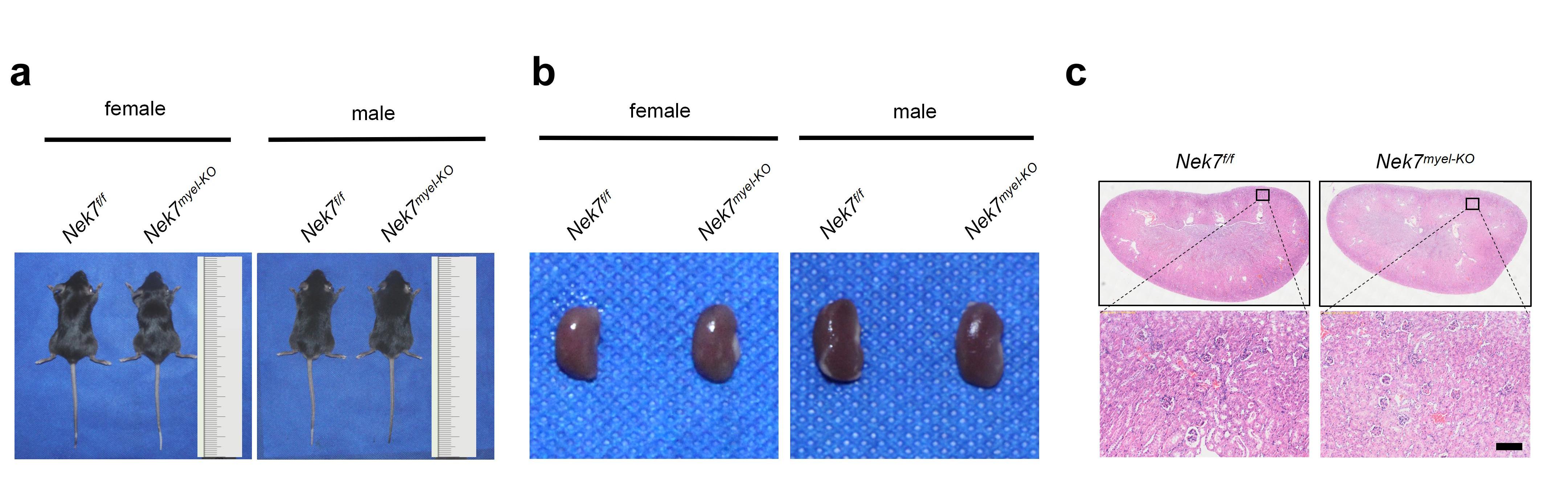
**

**The phenotyping of Nek7^myel-KO^ mice. a**, The appearance and development of *NEK7^f/f^* and *NEK7^myel-KO^* mice. Scale bar, 20cm. **b,** The morphology of the kidneys of *NEK7^f/f^* and *NEK7^myel-KO^* mice. **c**, Representative images of HE staining of the renal tissue sections from *NEK7^f/f^* and *NEK7^myel-KO^* mice. Scale bar, 100μm.

Table. S1.

| Table 1: ATIBODIES | | |
| --- | --- | --- |
| ANTIBODIES | SOURCE | IDENTIFIER |
| A20 | Cell Signaling Technology | Cat#5630S |
| NLRP3 | Cell Signaling Technology | Cat#15101S |
| ɑ-TUBULIN | Proteintech | Cat#11224-1-AP |
| Goat anti-Rabbit IgG | Signaling Antibody | Cat#L3012 |
| Goat anti-Mouse IgG | Signaling Antibody | Cat#L3032 |
| CD11b-conjugated-FITC-antibody | BD Biosciences | Cat#561688 |
| F4/80-conjugated-PE-antibody | BD Biosciences | Cat#565410 |
| CASPASE1 | Cell Signaling Technology | Cat#89332S |
| IRF3 | Cell Signaling Technology | Cat#4302S |
| Phospho-IRF3 | Cell Signaling Technology | Cat#29047S |
| TBK1 | Abcam | Cat#ab40676 |
| Phospho-TBK1 | Cell Signaling Technology | Cat#5483S |
| NF-κB p65 | Cell Signaling Technology | Cat#8242T |
| phospho-NF-κB p65 | Cell Signaling Technology | Cat#3033 |
| CASPASE3 | ZEN BIO | Cat#252555 |
| cleaved-CASPASE3 | ZEN BIO | Cat#341052 |
| STAT1 | Abcam | Cat#ab109320 |
| Phospho-STAT1 | Cell Signaling Technology | Cat#9171S |
| MLKL | ZEN BIO | Cat#513451 |
| Phospho-MLKL | ZEN BIO | Cat#530949 |
| GSDMD | Abcam | Cat#ab209845 |
| NEK7 | Abcam | Cat#ab133514 |
| Ubiquitin | Santa Cruz | Cat#sc-8017 |
| HA | Cell Signaling Technology | Cat#3724S |
| Flag-HRP | Sigma | Cat#A8592 |

Table. S2

| Table 2: primer sequences for PCR | | |
| --- | --- | --- |
| Primers |  | Sequences (5’–3’) |
| *mGapdh* | Forward | TGTGTCCGTCGTGGATCTGA |
| *mGapdh* | Reverse | TTGCTGTTGAAGTCGCAGGAG |
| *mTnfaip3* | Forward | CTCGGAACTTTAAATTCCGC |
| *mTnfaip3* | Reverse | GGGTAAGTTAGCTTCATCC |
| *mpro-IL-1β* | Forward | GAAATGCCACCTTTTGACAGTG |
| *mpro-IL-1β* | Reverse | TGGATGCTCTCATCAGGACAG |
| *mNlrp3* | Forward | TCACAACTCGCCCAAGGAA |
| *mNlrp3* | Reverse | AAGAGACACCAGGAGCTAG |
| *mNek7* | Forward | TCTTGGATGGAGTGCCGGTA |
| *mNek7* | Reverse | TCTCAGGGATTAGCCTCTTTTGT |
| *mCxcl10* | Forward | CCTGCAGGATGATGGTCAAG |
| *mCxcl10* | Reverse | GAATTCTTGCTTCGG CAGTT |
| *mIfn-β* | Forward | TCCGAGCAGAGATCTTCAGGAA |
| *mIfn-β* | Reverse | TGCAACCACCACTCATTCTGAG |
| *mTnf-α* | Forward | TCTTCTCATTCCTGCTTGTGG |
| *mTnf-α* | Reverse | GGTCTGGGCCATAGAACTGA |
| *mIl-6* | Forward | GATGGATGCTACCAAACTGGAT |
| *mIl-6* | Reverse | CCAGGTAGCTATGGTACTGCAGA |
| *hGAPDH* | Forward | AGCCACATCGCTCAGACAC |
| *hGAPDH* | Reverse | GCCCAATACGACCAAATCC |
| *Geno-mTnfaip3* | Forward | AAGCTAGGAGAGGAAAATCCCAG |
| *Geno-mTnfaip3* | Reverse | GCTAAGGCCTTGATACCACTATTC |
| *Geno-mNek7^myel-KO^* | Forward | AAGACTAAGTCCTATGTGCTAGGC |
| *Geno-mNek7^myel-KO^* | Reverse | CTTCAAGTCTGACATCTGGTCTCTTGG |
| *Cre* | Forward | CCCAGAAATGCCAGATTACG |
| *Cre* | Reverse | CTTGGGCTGCCAGAATTTCTC |
